# Bromine Pentafluoride BrF_5_, the Formation of [BrF_6_]^−^ Salts, and the Stereochemical (In)activity of the Bromine Lone Pairs

**DOI:** 10.1002/chem.202202466

**Published:** 2022-10-31

**Authors:** Martin Möbs, Tim Graubner, Kim Eklund, Antti J. Karttunen, Florian Kraus

**Affiliations:** ^1^ Anorganische Chemie, Fluorchemie Philipps-Universität Marburg Hans-Meerwein-Str. 4 35032 Marburg Germany; ^2^ Department of Chemistry and Materials Science Aalto University 00076 Aalto Finland

**Keywords:** bromine pentafluoride, crystal structure, hexafluoridobromate, photochemistry, quantum-chemical calculations

## Abstract

BrF_5_ can be prepared by treating BrF_3_ with fluorine under UV light in the region of 300 to 400 nm at room temperature. It was analyzed by UV‐Vis, NMR, IR and Raman spectroscopy. Its crystal structure was redetermined by X‐ray diffraction, and its space group was corrected to *Pnma*. Quantum‐chemical calculations were performed for the band assignment of the vibrational spectra. A monoclinic polymorph of BrF_5_ was quantum chemically predicted and then observed as its low‐temperature modification in space group *P*2_1_/*c* by single crystal X‐ray diffraction. BrF_5_ reacts with the alkali metal fluorides *A*F (*A*=K, Rb) to form alkali metal hexafluoridobromates(V), *A*[BrF_6_] the crystal structures of which have been determined. Both compounds crystallize in the K[*A*sF_6_] structure type (*R* $\bar{3}$ , no. 148, *hR*24). For the species [BrF_6_]^+^, BrF_5_, [BrF_6_]^−^, and [IF_6_]^−^, the chemical bonds and lone pairs on the heavy atoms were investigated by means of intrinsic bond orbital analysis.

## Graphical abstract

**Taking a closer look**: We present the photochemical synthesis of BrF_5_, its high‐ and low‐temperature crystal structures, its reactions with some alkali metal fluorides and a discussion of the free valence electron pairs on the bromine atoms of the BrF_5_ molecules and [BrF_6_]^−^ anions by using intrinsic bond orbitals.

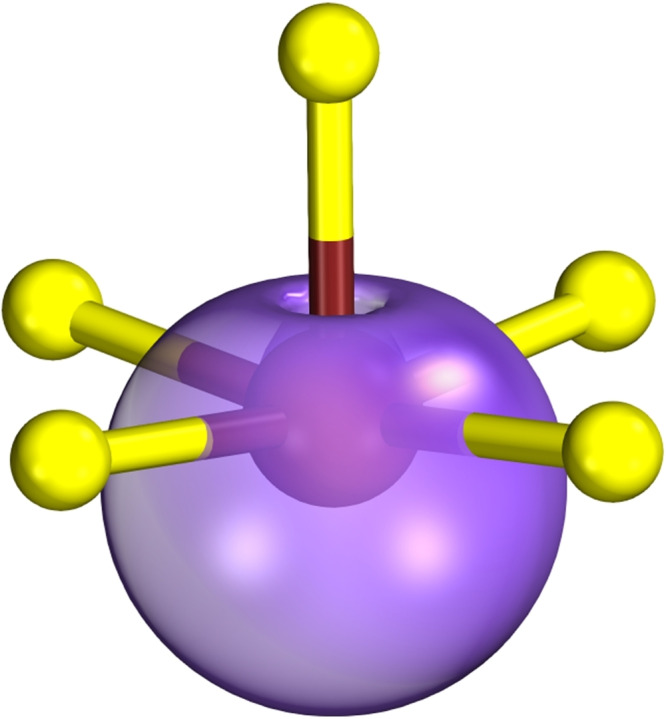

## Introduction

Bromine pentafluoride is one of the most reactive halogen fluorides, probably outperformed in its reactivity only by ClF_3_.[^1^, ^2^, ^3^, ^4^, ^5^] BrF_5_ was discovered by Ruff and Menzel in 1931 and described as a colorless liquid that freezes at 211.85 K (−61.30 °C) and boils at 313.65 K (+40.50 °C).^[6]^ BrF_5_ is the highest known binary fluoride of bromine. The existence of the hypothetical BrF_7_ molecule has not yet been proven as attempts of a further oxidation of BrF_5_ resulted in the formation of the [BrF_6_]^+^ cation known for the compounds [BrF_6_][AsF_6_] and [BrF_6_][Sb_2_F_11_].^[7]^

The conventional synthesis of BrF_5_ ^[6]^ takes place by reacting fluorine gas with gaseous BrF_3_ at 473 K in a platinum or copper vessel [Eq. 1].

(1) $$\mathrm{BrF}{}_{3}+F{}_{2}\overset{473\;K}{\underset{}{\to }}\mathrm{BrF}{}_{5}$$

The pale yellow (red if more heavily contaminated by mixtures of other bromine fluorides or Br_2_) crude BrF_5_ is then fractionally distilled for purification.^[6]^ Another synthetic route for the preparation of BrF_5_ is the fluorination of KBr, as described by Hyde and Boudakian.^[8]^ Contact with moisture or the use of unsuitable or insufficiently passivated vessel materials will also cause BrF_5_ to take on a pale yellow to deep red color.^[6]^ In this case, fluorine can be passed through the liquid at room temperature until all the Br_2_ and BrF_3_ has reacted.[^7^, ^9^] After degassing, BrF_5_ can be distilled onto dry NaF for storage, that reacts with traces of HF and BrF_3_.^[9]^ Pure BrF_5_ is colorless.^[6]^

Compounds such as BrF_5_ are hard to come by or simply unobtainable for academic research purposes, therefore we looked for a simple method for their synthesis at room temperature on a laboratory scale. While the photosynthesis of ClF_5_ had already been reported in various sources,[^10^, ^11^, ^12^] to the best of our knowledge no methods are yet known for the photochemical preparation of BrF_5_. However, the photochemical fluorination of bromine and BrF_3_, respectively, to BrF_5_ should be straightforward, since the ionization energies of bromine are even lower than those of chlorine.

Due to the lower temperature of the synthesis presented here compared to the traditional routes, no or significantly less wall reactions with the vessel materials take place. Also, the decomposition of BrF_5_ at higher temperatures into BrF_3_ and F_2_ is suppressed.^[1]^ Therefore, BrF_5_ is obtained in nearly quantitative yield and high purity.

The crystal structure of solid BrF_5_ was determined in 1957 by Burbank and Bensey,^[13]^ but due to the data quality a redetermination of the crystal structure was performed here. We present a modified structure model for HT‐BrF_5_, a novel low‐temperature modification LT‐BrF_5_, reactions of BrF_5_ with KF and RbF, and discuss the effect of the lone pair on the Br atom within the BrF_5_ molecule and the [BrF_6_]^−^ anion in comparison to other species.

## Results and Discussion

**Scheme 1:**
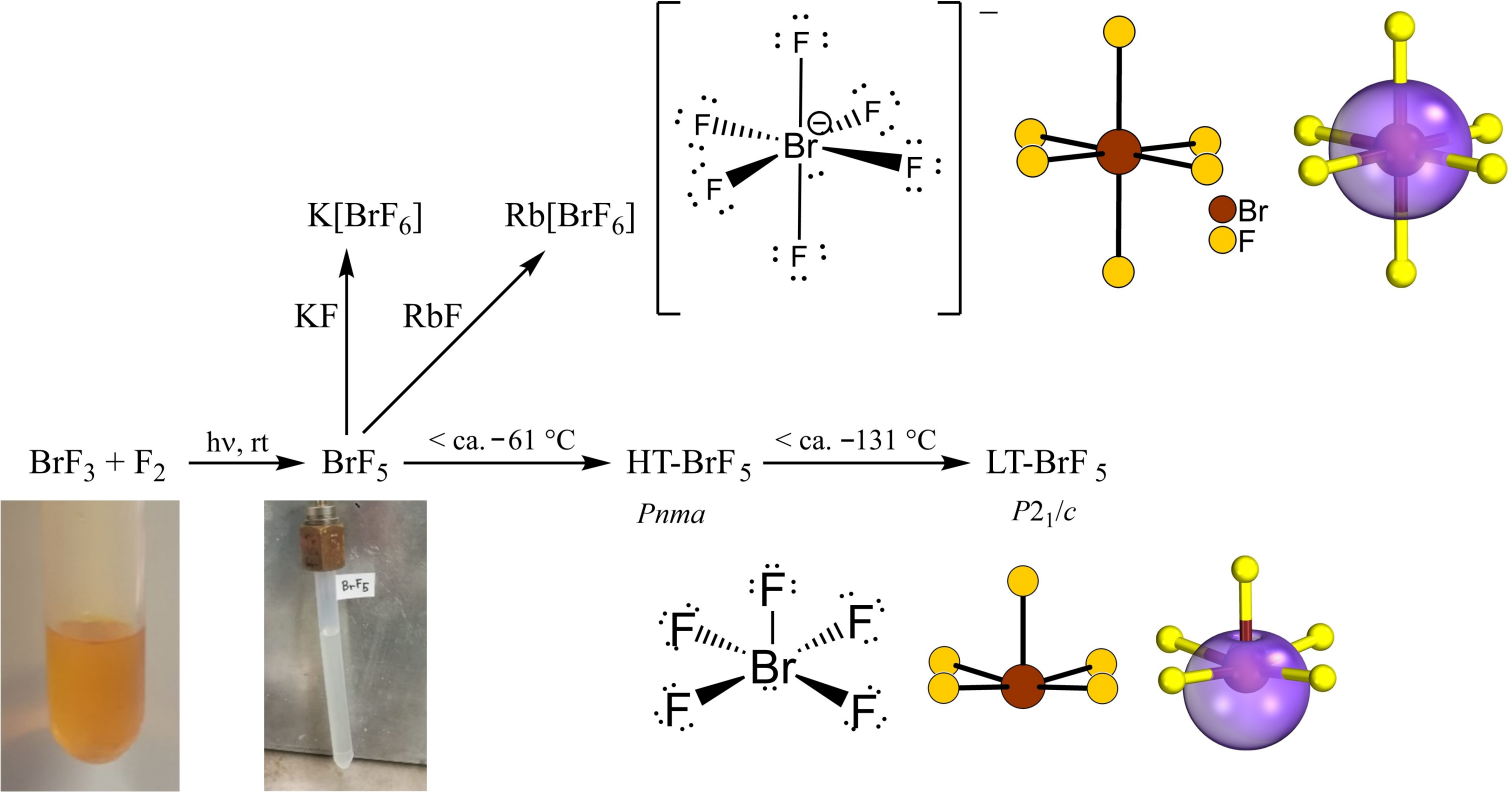
A summary of the works reported here. Preparation of BrF_5_, solid‐state structures of its high‐temperature (HT) and low‐temperature (LT) polymorph, its reactions with the alkali metal fluorides KF and RbF. Photographs of BrF_3_ and BrF_5_, as well as the Lewis structures of the BrF_5_ molecule and the [BrF_6_]^−^ anion, sections of the crystal structures, and the lone pairs on the Br atoms using IBOs are shown.

Scheme 1 provides an overview of the works reported in this manuscript, the photochemical synthesis of BrF_5_, its high‐temperature and low‐temperature crystal structures, its reactions with some alkali metal fluorides and a discussion of the free valence electron pairs on the Br atoms of BrF_5_ molecules and [BrF_6_]^−^ anions by using intrinsic bond orbitals (IBOs).

### Photochemical preparation of bromine pentafluoride

BrF_5_ was obtained by irradiation of BrF_3_ and F_2_ with UV light at room temperature according to Equation 2.

(2) $$\mathrm{BrF}{}_{3}+F{}_{2}\overset{h\nu ,\;\mathrm{RT}}{\underset{}{\to }}\mathrm{BrF}{}_{5}$$

The reaction can be performed either with pure fluorine gas or gas diluted with argon. The use of diluted fluorine does not entail any major disadvantages, apart from the smaller amount of F_2_ being present for BrF_5_ synthesis at the same overall pressure. Pure BrF_5_ could be obtained at irradiation wavelengths between 300 and 400 nm in both cases. The product was characterized by X‐ray diffraction, NMR, UV‐Vis, Raman and IR spectroscopy and by reaction with alkali metal fluorides.

### Selection of UV lamps of suitable wavelength

**Figure 1:**
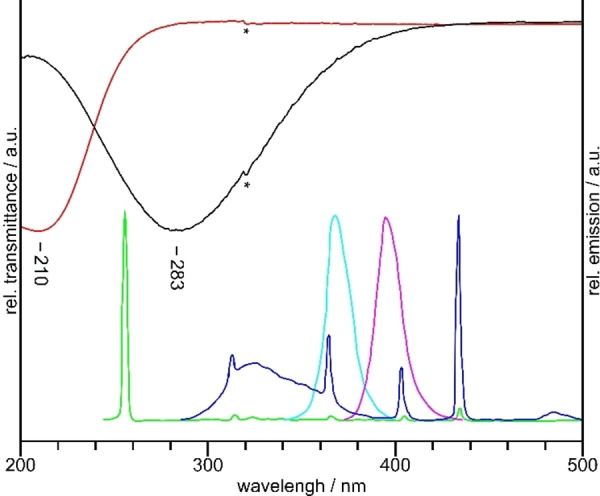
UV‐Vis transmittance spectra of BrF_5_ (red) and F_2_ (black) as well as emission spectra of the UV lamps used: Osram Puritec HNS UV−C,^[14]^ 254 nm (green), ExoTerra Reptile UVB200^[15]^ (blue), Nichia NVSU233B,^[16]^ 365 nm (cyan), Osram LuxiGen, 395 nm (magenta).^[17]^ An artifact due to the lamp change of the UV‐Vis spectrometer at 320 nm is marked by an asterisk. Data for the emission spectra were extracted from the literature[^14^, ^15^, ^16^, ^17^] by using the Engauge Digitizer software.^[18]^

Various UV lamps of different wavelengths were tested for the photochemical synthesis of BrF_5_. Their emission spectra are shown in Figure 1. BrF_5_ is formed independent of the used UV lamp, however, it was not possible to achieve complete conversion when the low‐pressure mercury vapor lamp with a peak wavelength of 254 nm was used. Instead, yellowish samples were obtained which additionally contained BrF_3_, as was shown by Raman spectroscopy. The use of the other lamps with emission maxima at longer wavelengths resulted in all cases in pure, colorless BrF_5_. This leads to the conclusion that BrF_5_ is photodissociated due to its self‐absorption at low wavelength and an equilibrium between BrF_5_ and BrF_3_ is established. However, as demonstrated by the transmission spectra of BrF_5_ and F_2_ in Figure 1, their absorption maxima are separated well enough, so that at higher wavelengths fluorine molecules can be photo‐dissociated selectively.

### NMR spectroscopy

**Figure 2:**
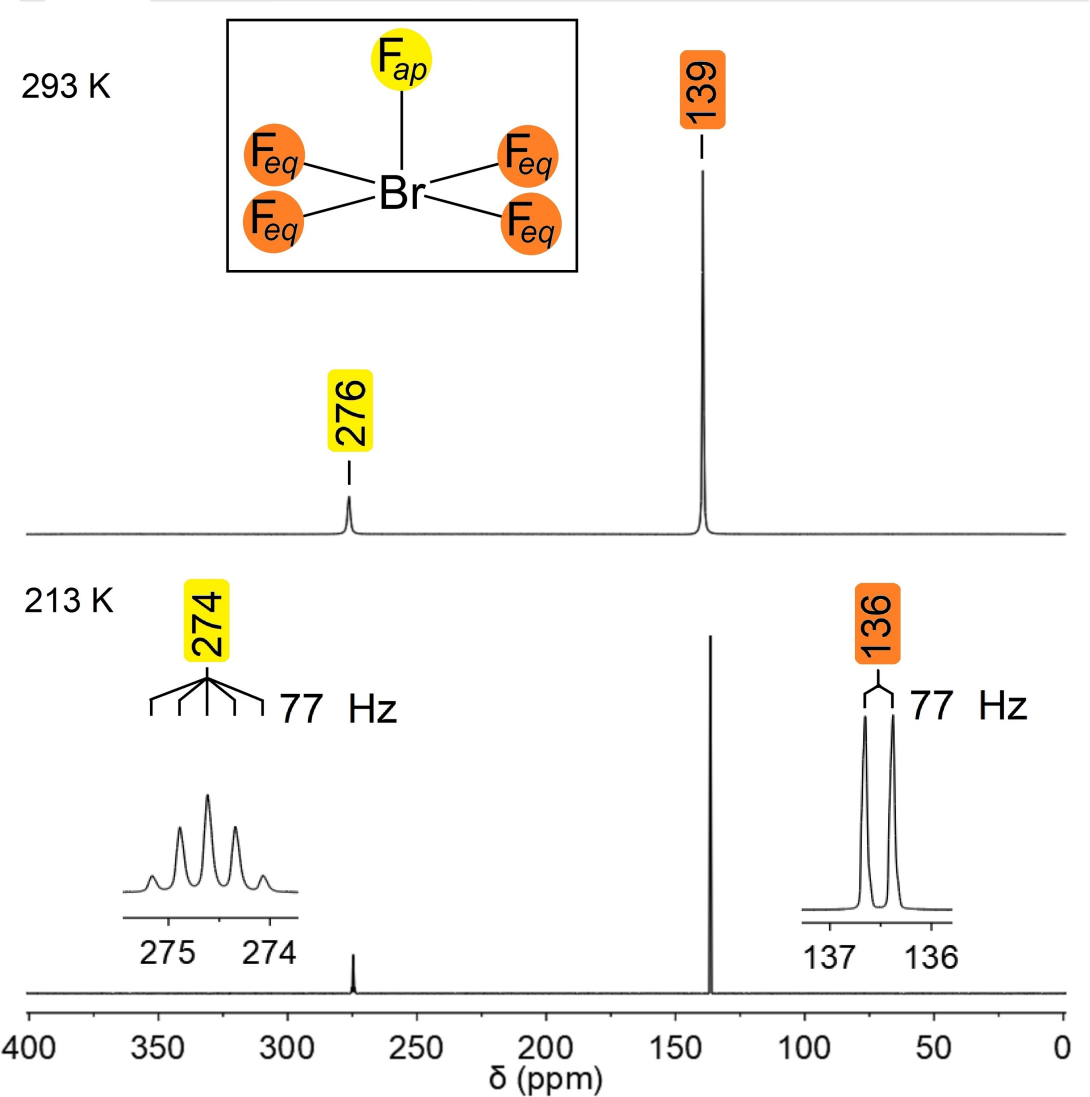
^19^F NMR spectra of neat BrF_5_ at room temperature (top) and at 213 K (bottom), slightly above the melting point. The assignment of the observed signals to the F atoms is indicated in yellow (F_ap_) and orange (F_eq_).

^19^F NMR spectra of the as‐obtained BrF_5_ were recorded at room temperature and at 213 K and are shown in Figure 2. Two signals with an integral ratio of 1 : 4 are present and assigned to the one apical, F_ap_, and the four equatorial, F_eq_, fluorine atoms of BrF_5_. Spectra recorded at 273 K or below show a splitting of the two signals into a quintet and a doublet due to ^2^ *J*(^19^F,^19^F) coupling. Thus, assignments of the signals with a chemical shift of around 276 ppm to the F_ap_ atom and the one at around 139 ppm to the F_eq_ atoms are evident. The obtained spectra are in agreement with the literature.^[19]^ NMR spectra and data obtained from variable temperature measurements from 213 to 300 K are summarized in Figure S1 and Table S1 in the Supporting Information. NMR chemical shifts calculated at the CCSD(T)/cc‐pwCVTZ level of theory agree with the experimental findings (see the Experimental Section for computational details). The chemical shift for the F_ap_ atom was calculated to be at 277 ppm and the one for the F_eq_ atoms at 138 ppm.

### Vibrational spectroscopy

**Figure 3:**
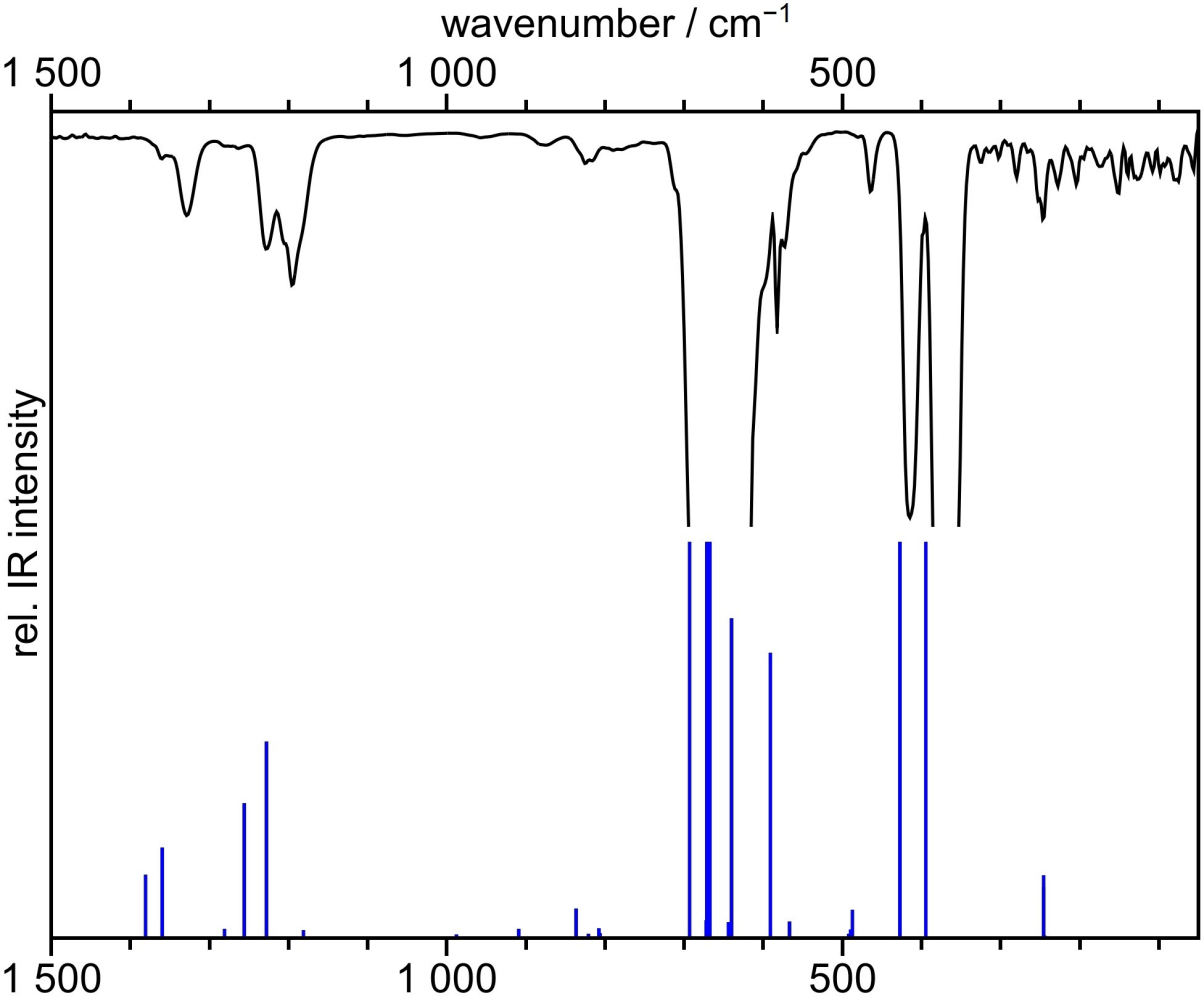
IR spectrum of gaseous BrF_5_ at ca. 125 mbar (black line). The corresponding calculated and intensity‐scaled IR bands are shown as blue lines, of which the strongest were cut at the same arbitrary value. In the region above 1500 cm^−1^ and up to 4000 cm^−1^, no additional bands were observed or calculated.

IR spectra of gaseous BrF_5_ were recorded at room temperature at various pressures (Figures S6 and S7) in order to resolve strong fundamental vibrations as well as weaker combination modes. A spectrum together with the quantum chemically calculated modes is shown in Figure 3. The quantum‐chemical calculation for band assignment of the fundamental and combination modes was performed at the CCSD(T)/cc‐pVTZ level of theory.

**Figure 4:**
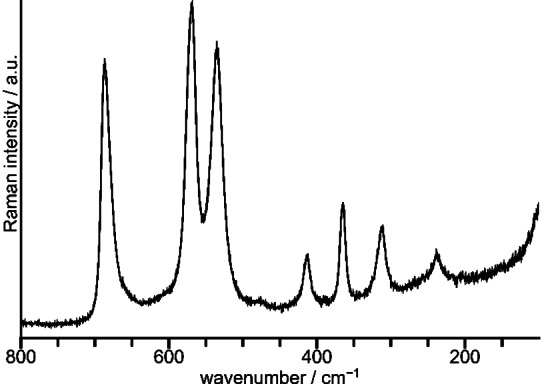
Raman spectrum of liquid BrF_5_ at room temperature. No additional bands were present in the region above 800 cm^−1^ and up to 4000 cm^−1^.

The Raman spectrum shown in Figure 4 was recorded for liquid BrF_5_. For practical reasons, no IR spectrum could be acquired on liquid BrF_5_. Both the Raman as well as the gas‐phase IR spectrum are in agreement with those given in the literature.[^20^, ^21^] The tetragonal‐pyramidal BrF_5_ molecule (*C*_4*v*_) shows twelve normal modes of vibration. Three modes are doubly degenerate, six are non‐degenerate. These are assigned into the irreducible representations *A*_1_, *B*_1_, *B*_2_, and *E* of the point group. All these vibrations are Raman‐active, whereas only the *A*_1_ and *E* vibrations are IR‐active. The band assignment for the fundamental vibrations is given in Table 1. Two of the active Raman frequencies, *ν*_5_ and *ν*_7_, were not observed in the recorded spectrum. Based on the calculated bands, it can be assumed that *ν*_5_ overlaps with *ν*_9_, and *ν*_7_ most likely coincides with *ν*_1_, leading to its slightly asymmetric shape. The numbering of the vibrations follows the literature.^[22]^

**Table 1:** Observed fundamental vibration frequencies of BrF_5_ and band assignment. The IR spectrum was recorded on gaseous BrF_5_, the Raman spectrum on liquid BrF_5_.

Assignment	IR	Raman	Description
	frequency [cm^−1^]		
ν_1_, *A*_1_	683	687	stretching vibration ν(Br−F_ap_)
ν_2_, *A*_1_	582	569	symmetric stretching vibration ν_s_(Br−F_eq_)
ν_3_, *A*_1_	369	365	out‐of‐plane bending π(Br−F_eq_)
ν_4_, *B*_1_	–	535	antisymmetric stretching ν_s_(Br−F_eq_)
ν_5_, *B*_1_	–	n.o.	antisymmetric deformation δ_a_(Br−F_eq_)
ν_6_, *B*_2_	–	312	symmetric in‐plane bending δ_s_(Br−F_eq_)
ν_7_, *E*	646	n.o.	degenerate stretching ν_d_(Br−F_eq_)
ν_8_, *E*	414	414	degenerate out‐of‐plane bending π_d_(Br−F_eq_)
ν_9_, *E*	247	238	degenerate in‐plane bending δ_d_(Br−F_eq_)

A more comprehensive band assignment for the IR spectrum including the combination bands is given in Table S4. Raman spectra of liquid and solid BrF_5_ at various temperatures are shown in Figures S2–S5, their band assignments in Tables S2 and S3. Calculated Raman spectra of the two crystalline modifications are also available in Figures S8 and S9.

### Handling and reactivity

In several cases, especially during our attempts to record IR spectra on BrF_5_, we visually observed a color change of BrF_5_ gas from colorless to yellowish‐brown, which indicated the decomposition of some traces of BrF_5_. The nature of the yellowish‐brown species is still under investigation. Even before the color change is visible to the eye, additional IR bands appear in the region above 3690 cm^−1^ corresponding to the rotation‐vibration bands of HF. This decomposition of BrF_5_ does not occur when all surfaces that come in contact with it are well passivated, thoroughly baked out and as free of moisture as possible. In order to achieve this, steel apparatuses are usually baked out in a hot air bath at about 873 K in vacuo. Because it is technically not possible to bake out our IR gas cell completely, which is due to the limited thermal stress resistance of the optical windows, we suspect traces of water being the reason for the decomposition of BrF_5_. To overcome this issue, we first filled the gas cell with BrF_5_ or ClF_3_ so that they react with “all” the moisture, then pumped of the volatiles using a vacuum of circa 10^−3^ mbar and finally filled the cell with fresh BrF_5_ for the measurement.

When BrF_5_ is stored in vessels made out of fluoropolymers like PFA or FEP, HF impurities are also present after some days. Both BrF_5_ and moisture dissolve and diffuse through the walls of the fluoropolymer vessel so that the contamination increases over time. That is why vessels made out of passivated stainless steel, nickel or Monel should be preferred for storage and handling. When fluoropolymers are required because of their translucency, the vessels can be baked out in vacuum and then flushed with fluorine several times. This “saturates” the polymer with F_2_ and temporarily displaces other contaminants such as H_2_O dissolved in the fluoroplastic.

### Crystal structure of HT‐BrF_5_

The crystal structure of BrF_5_ was first published in 1957 by Burbank and Bensey.^[13]^ According to them, BrF_5_ crystallizes in the orthorhombic space group *Cmc*2_1_ (36) at 153 K. However, our diffraction data show reflections that clearly violate the extinction condition of the *C*‐centering. These violations were observed not only in a powder diffraction pattern of BrF_5_ recorded at 180 K, see below, but also in the single‐crystal X‐ray diffraction data acquired at temperatures of 200, 180, 150 and 100 K.

Below its melting point of 211.85 K (−61.3 °C), BrF_5_ crystallizes in space group *Pnma* (62) but undergoes a phase transition at about 142 K as shown by variable temperature powder X‐ray diffraction, see the Supporting Information. At 130 K we observed the formation of a monoclinic low‐temperature (LT) modification, crystallizing in space group *P*2_1_/*c* (14). The first frames collected during the X‐ray diffraction experiment at this temperature showed still only the orthorhombic polymorph, then both modifications were observed, while the last frames contained only reflections of LT‐BrF_5_. A dataset containing only the reflections of LT‐BrF_5_ was collected at 100 K. Selected crystallographic data and details of the structure determinations are given in Table 2.

**Table 2:** Selected crystallographic data and details of the structure determinations of BrF_5_ at various temperatures.

		HT‐BrF_5_		LT‐BrF_5_
Formula	BrF_5_			
Molar mass [g ⋅ mol^−1^]	174.91			
Space group (no.)	*Pnma* (62)			*P*2_1_/*c* (14)
*a* [Å]	7.8447(13)	7.841(3)	7.8291(3)	6.3355(4)
*b* [Å]	6.4538(12)	6.415(3)	6.3861(2)	7.2166(4)
*c* [Å]	7.3062(12)	7.261(4)	7.2364(2)	7.7803(5)
*β* [°]	90	90	90	94.255(3)
*V* [Å^3^]	369.90(11)	365.2(3)	361.80(2)	354.74(4)
*Z*	4	4	4	4
Pearson symbol	*oP*24	*oP*24	*oP*24	*mP*24
*ρ*_calc._ [g ⋅ cm^−3^]	3.141	3.181	3.211	3.275
*μ* [mm^−1^]	11.092	11.235	11.340	11.566
Color	colorless	colorless	colorless	colorless
Crystal morphology	needle	needle	needle	needle
Crystal size [mm^3^]	0.784×0.274×0.248	0.665×0.299×0.285	0.784×0.274×0.248	0.665×0.299×0.285
*T* [K]	200	180	150	100
*λ* [Å] (Mo_Kα_)	0.71073			
No. of reflections	10630	14703	11267	1286
*θ* range [°] (min, max)	3.811, 31.498	3.825, 33.713	2.602, 39.194	3.224, 32.568
Range of Miller indices	−11≤*h*≤11,	−12≤*h*≤12,	−11≤*h*≤11,	−9≤*h*≤9,
	−9≤*k*≤9,	−10≤*k*≤10,	−9≤*k*≤9,	0≤*k*≤10,
	−10≤*l*≤10	−11≤*l*≤11	−10≤*l*≤10	0≤*l*≤11
Absorption correction	multi‐scan			
*T*_max_, *T*_min_	1.0000, 0.2064	0.1954, 0.0250	0.1954, 0.0250	0.1577, 0.0262
*R*_int_, *R*_*σ*_	0.0391, 0.0140	0.0515, 0.0251	0.0351, 0.0132	0.0677, 0.0180
Completeness of the data set	0.994	0.999	0.994	0.998
No. of unique reflections	663	781	674	1286
No. of parameters	34	34	34	55
No. of restraints	0	0	0	0
No. of constraints	0	0	0	0
*S* (all data)	1.219	1.153	1.283	1.047
*R*(*F*) (*I*≥2*σ*(*I*), all data)	0.0339, 0.0404	0.0315, 0.0392	0.0291, 0.0325	0.0337, 0.0371
*wR*(*F* ^*2*^) (*I*≥2*σ*(*I*), all data)	0.0801, 0.0837	0.0732, 0.0783	0.0715, 0.0732	0.0799, 0.0828
Largest diff. peak/hole [e Å^−3^]	0.850/−0.490	0.599/−0.445	0.435/−0.482	1.609/−0.619

We solved and refined the crystal structure of the high‐temperature (HT) polymorph in space group *Pnma* (62). Attempts to refine the crystal structure in the non‐centrosymmetric subgroup *Pna*2_1_ also lead to the essentially same structure model, however the Flack parameter of 0.46(7), the ADDSYM[^23^, ^24^] algorithm implemented in the PLATON^[25]^ software, as well as strong correlation of atomic coordinates clearly suggested the centrosymmetric space group *Pnma* to be the superior choice.

**Figure 5:**
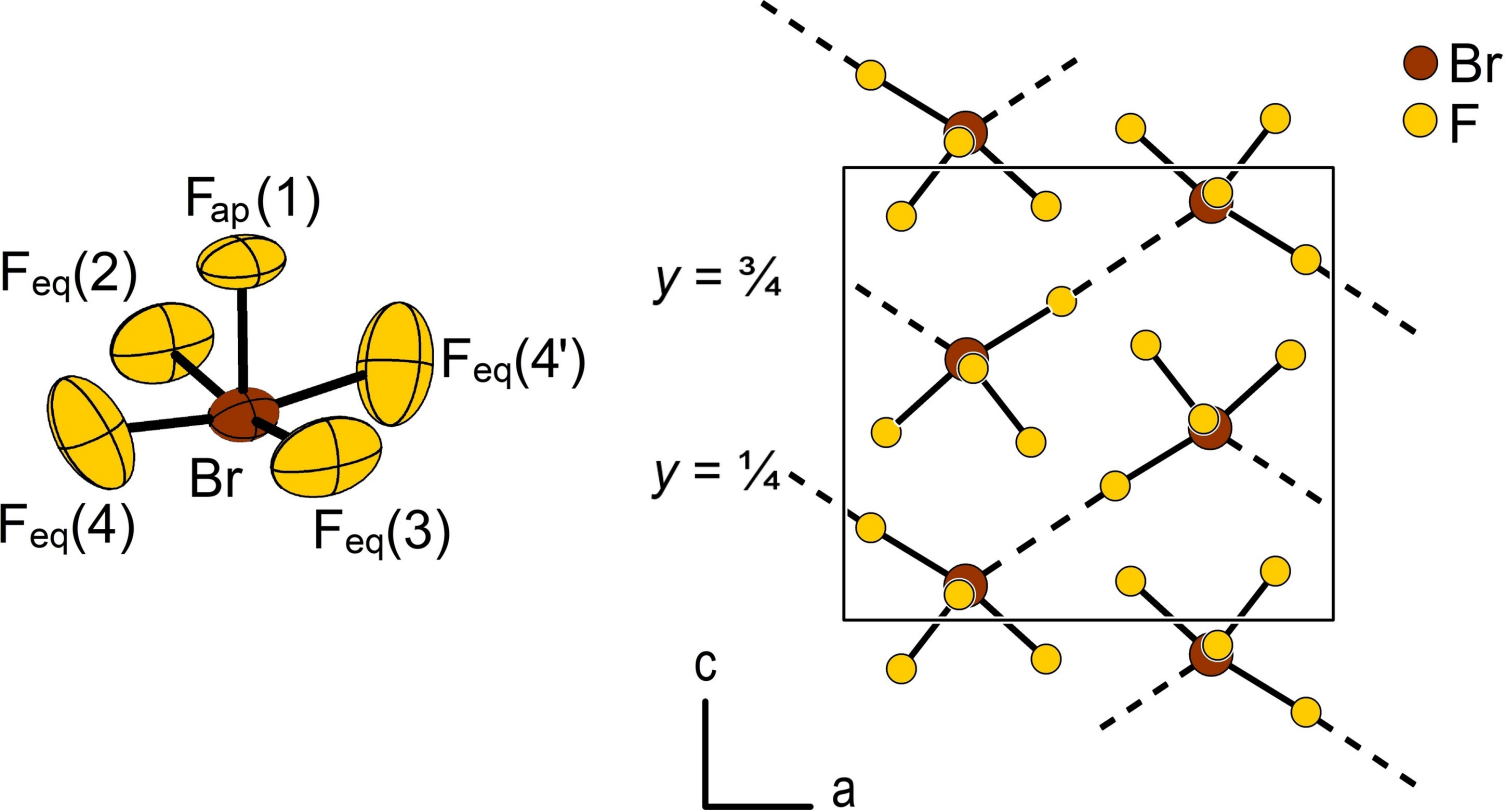
Left: Section of the crystal structure of BrF_5_ showing the square pyramidal molecular shape. Displacement ellipsoids are shown at 70 % probability at 150 K. Right: Projection of the crystal structure showing the infinite one‐dimensional strands of interconnected BrF_5_ molecules running parallel to the *a*‐axis. Atoms are shown isotropic with arbitrary radii. Symmetry transformation for the generation of the F(4’) equivalent atom: *x*, 1/2 −*y*, *z*.

The following description of the crystal structure refers to the diffraction data collected at 150 K, allowing for a direct comparison with the previous structure model. In the solid state, BrF_5_ adopts a square pyramidal molecular structure (Figure 5), as already indicated by NMR and vibrational spectroscopy for the liquid and gas phase. The bromine atom resides on Wyckoff position 4*c* (.*m*.) and is surrounded by the F_ap_(1) atom (4*c*, .*m*.), the F_eq_ atoms F(2) and F(3) on positions 4*c* (.*m*.), and the F_eq_(4) atom on the 8*d* (1) position. The Br atom is not located within the base of the pyramid, but lies with 0.1726(18) Å outside a least‐squares plane spanned by the four F_eq_ atoms, showing the putative space requirement of the lone pair of the Br atom. The bond angles between the apical and equatorial fluorine atoms F_ap_−Br−F_eq_ are therefore smaller than 90 ° and are 83.75(11)°, 2×84.16(10)°, and 85.36(14)°. The same phenomenon is visible in the previously reported structure model, where the distance between the Br atom and the base of the pyramid was reported with 0.1734 Å (with no esds given in literature).^[13]^ The respective F_ap_−Br−F_eq_ angles diverge much more with 80.4°, 2×85.4°, and 86.5°.

The Br−F_eq_ bonds range from 1.741(2) to 1.777(2) Å and are therefore slightly longer than the Br−F_ap_ bond with 1.678(2) Å. The Br−F_eq_ bond lengths of the previously reported structure show a larger deviation from the mean value with 1.7460 to 1.8206 Å, which can be attributed to the choice of the space group. However, the reported Br−F_ap_ distance agrees with 1.6784 Å with the value redetermined here.

An intermolecular interaction is present between the Br atoms and F_eq_ atoms of neighboring BrF_5_ molecules with a Br⋅⋅⋅F_eq_ distance of 2.880(2) Å. As expected, the F_eq_ atom involved in the intermolecular interaction is the one with the longest intramolecular Br−F_eq_ distance of 1.777(2) Å. The planes spanned by the four equatorial fluorine atoms of each of two adjacent BrF_5_ molecules intersect at an angle of 106.42(6)°. As a result of this intermolecular interaction, flat, zigzag‐like chains are formed running parallel to the *a*‐axis. The crystal structure of BrF_5_ can therefore be described with the Niggli formula ${}_{\infty }^{1}\left[{\mathrm{BrF}}_{\frac{4}{1}}F_{\frac{2}{1+1}}\right]$ . To the best of our knowledge, the crystal structure of BrF_5_ represents a new structure type. The formation of oligomeric molecular structures, as known for other pentafluorides such as MoF_5_ (*mS*48), that is rings of Mo_4_F_20_, RuF_5_ (*mP*48), differently shaped Ru_4_F_20_ rings, or AuF_5_ (*oP*48), Au_2_F_10_ dimers, does not occur.[^26^, ^27^, ^28^] Similarity exists between the structures of BrF_5_ (*oP*24) and BrF_3_ (*oS*16) .^[29]^ As BrF_5_, BrF_3_ also forms one‐dimensional infinite strands with a flat zigzag‐like shape in its solid state. Thus, the bromine atoms are coordinated in a kite shape by F atoms and have a coordination number of 3+1. The intramolecular Br−F distances in the BrF_3_ structure are in between 1.71(1) and 1.888(9) Å and therefore are in average longer than in the BrF_5_ molecule, as expected for bromine in oxidation state +III. The F atom with the longest intramolecular Br−F bond of 1.888(9) Å is the one that makes intermolecular contact with the neighboring BrF_3_ molecule. The intermolecular Br⋅⋅⋅F distance is 2.451(12) Å, and thus, more than 0.4 Å shorter than in the structure of BrF_5_. The shorter intermolecular Br⋅⋅⋅F distance is likely attributed to steric reasons.

**Figure 6:**
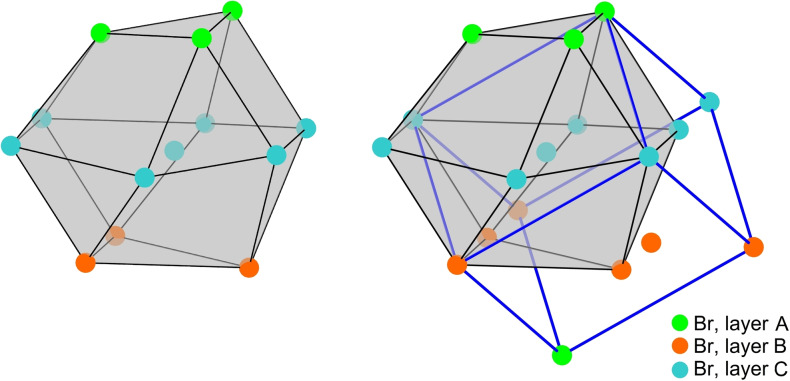
Left: Section of the crystal structure of BrF_5_ showing the cuboctahedral surrounding of a Br atom by nearest Br atoms. The green, blue and red Br atoms indicate the hexagonal layers of the cubic close packing. Right: Cuboctahedron of Br atoms and the relation of their arrangement to the pseudo‐F‐centered cell in blue. Bromine atoms are shown as spheres with arbitrary radii.

Looking at the arrangement of the Br atoms within the crystal structure of BrF_5_, a structural relation to the Cu type becomes obvious and a Bärnighausen‐tree is given in Figure S10. A Br atom is surrounded by 12 other Br atoms in the shape of a distorted cuboctahedron (Figure 6). The distortion that occurs is due to the formation of the chain motif adopted by the BrF_5_ molecules. This leads to a deviation from the pseudo‐face centering of the bromine atoms compared to the atom positions in the Cu type. In the setting chosen by Burbank and Bensey, this centering corresponds to the *C*‐centering in space group *Cmc*2_1_.

**Figure 7:**
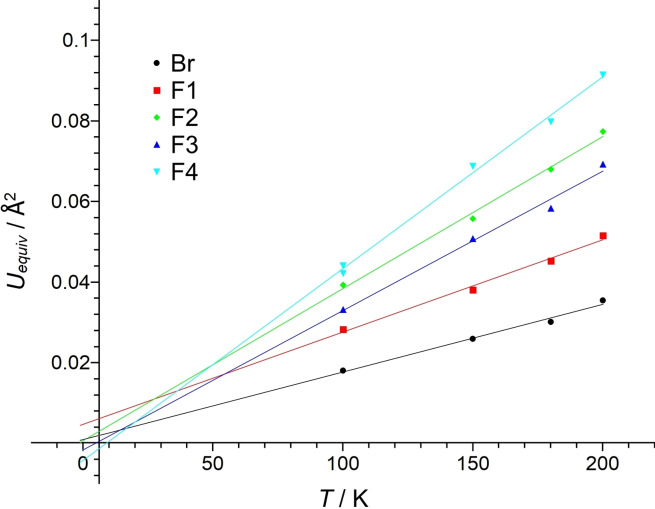
Plot of the equivalent displacement parameters vs. temperature. The displacement parameters observed at 100 K correspond to the structure of LT‐BrF_5_ and are therefore not directly comparable to those of HT‐BrF_5_. However, as the phase transition from HT‐ to LT‐BrF_5_ has no significant effect on the displacement parameters, we decided to use the data obtained at 100 K for extrapolation anyway.

The structure model proposed here in space group *Pnma* is reasonable, only the comparatively large displacement ellipsoids, especially of the F_eq_ atoms, may be unexpected. In order to examine whether these are physically meaningful and to be able to exclude that artificially large displacement parameters are obtained from a flawed structure model or an incorrect absorption correction, we recorded single‐crystal diffraction data at different temperatures. The obtained equivalent displacement parameters of the atoms were then plotted against the temperature (Figure 7). We observe that the extrapolated displacement parameters intersect the coordinate origin at 0 K within their standard uncertainties, implying that the strong atomic displacement is due to thermal effects indicating a “soft” molecular packing with weak intermolecular interactions.

**Figure 8:**
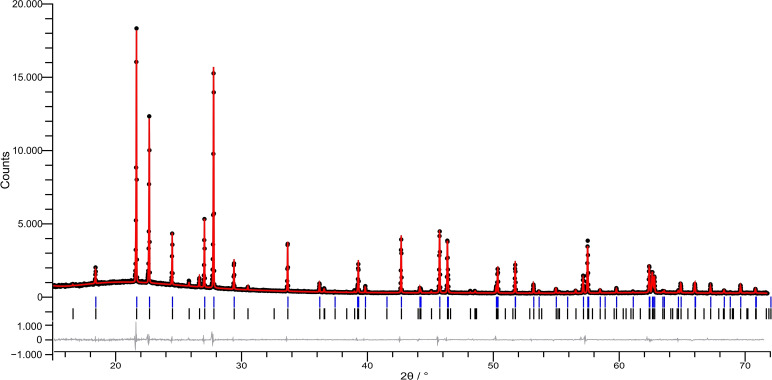
Powder X‐ray diffraction pattern of HT‐BrF_5_ recorded at 180 K. Measured data points are shown as black dots, calculated diffraction pattern as a result of the Rietveld refinement in red, and the difference curve is in gray. Vertical black bars indicate the calculated reflection positions for HT‐BrF_5_ crystallizing in space group *Pnma*; blue vertical bars indicate possible reflection positions considering the extinction conditions of the previously selected space group *Cmc*2_1_ for the old structure model. *Rp*=3.77, *Rwp*=5.42, *S*=1.295.

However, the powder X‐ray diffraction pattern of BrF_5_ recorded at 180 K (Figure 8) shows that the compound is diffracting rather well as sharp reflections are observed up to approximately 70° 2*θ* which indicates a proper three‐dimensional long‐range order of the BrF_5_ molecules within the crystal structure and that the thermal vibrations of the atoms are not severe at all.

All reflections could be assigned to the high‐temperature modification of BrF_5_ that crystallizes in space group *Pnma*. Space group *Cmc*2_1_, reported for the previous structure model,^[13]^ can be completely ruled out as several distinct reflections cannot be indexed (Figure 8). Details of the Rietveld refinement are available from Table S6.

### LT‐BrF_5_

**Figure 9:**
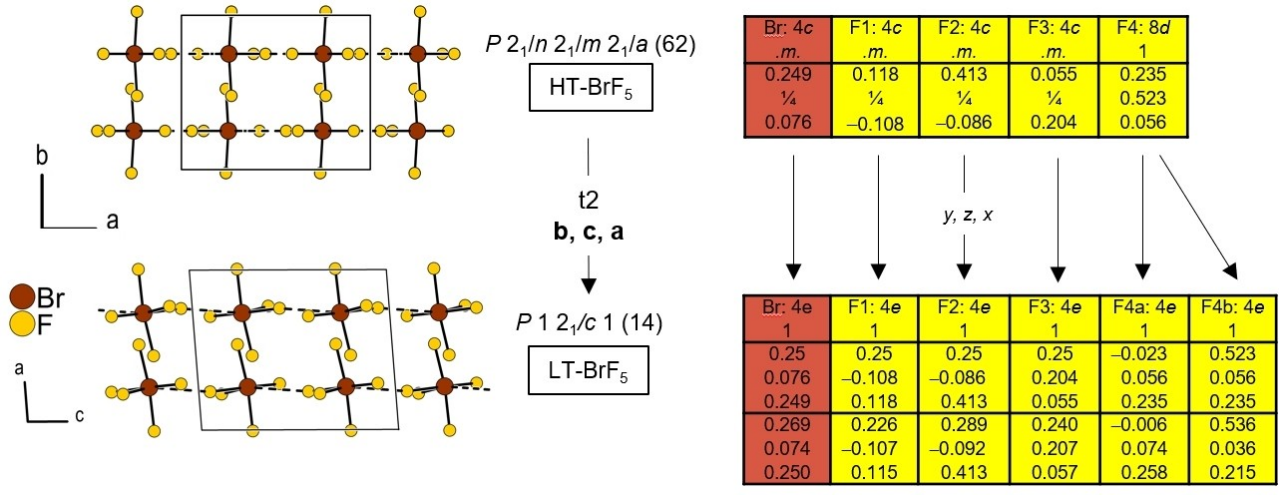
Left: Crystal structures of the high‐ and low‐temperature modifications showing the tilting of the BrF_5_ molecules within the 1D infinite strands as a result of the symmetry lowering. Right: Schematic representation of the symmetry relationship between the two modifications by means of a Bärnighausen tree.

The phase transition from HT‐ to LT‐BrF_5_ occurs at about 142 K, as shown by variable temperature powder X‐ray diffraction (Figure S11). The single‐crystal structure was recorded at 100 K. The shape of the BrF_5_ molecules as well as their interconnection to one‐dimensional infinite strands in the low‐temperature modification is similar compared to the high‐temperature polymorph. The Br−F_eq_ bond lengths range from 1.744(3) to 1.779(3) Å and are therefore identical within the standard uncertainties in comparison to the high‐temperature modification. The same applies to the Br−F_ap_ bond length measuring 1.686(2) Å and to the distances of the intermolecular Br⋅⋅⋅F_eq_ contacts measuring 2.881(3) Å. The Br atom is located 0.1707(16) Å below the least‐squares plane defined by the F_eq_ atoms. Thus, within the standard uncertainty this distance is also identical compared to the HT structure. The major structural difference between the two polymorphs is that in LT‐BrF_5_, together with the deviation of the monoclinic angle from 90°, the orientation of the BrF_5_ molecules within the zigzag‐strands also changes (Figure 9). The intrachain Br⋅⋅⋅Br distance shortens from 4.6555(3) Å in HT‐BrF_5_ to 4.6447(4) Å in LT‐BrF_5_. The angle by which the planes spanned by the F_eq_ atoms of two neighboring BrF_5_ molecules intersect decreases from 106.42(6)° in HT‐BrF_5_ to 103.17(7)° in LT‐BrF_5_. The shortest distance of two Br atoms in adjacent strands decreases from 4.8257(1) Å in HT‐BrF_5_ to 4.6489(4) Å in LT‐BrF_5_. The group‐subgroup relation of HT‐ and LT‐BrF_5_ is described in Figure 9 by means of a Bärnighausen tree.^[30]^

### Solid‐state quantum‐chemical calculations for BrF_5_

Quantum‐chemical calculations on BrF_5_ with dispersion‐corrected hybrid density functional method (DFT‐PBE0‐D3/TZVP), accounting for the weak intermolecular interactions, aided in the discovery of the low‐temperature modification. Calculations on the high‐temperature polymorph of BrF_5_ in space group *Pnma* showed a number of imaginary frequencies at 0 K, the largest being 33*i* cm^−1^. Following the largest imaginary mode allowed to predict the correct space group *P*2_1_/*c* and Wyckoff sequence of the low‐temperature modification of BrF_5_ even before the single‐crystal structure determination. A geometry optimization starting from the single‐crystal X‐ray structure of LT‐BrF_5_ (*P*2_1_/*c*) and a harmonic frequency calculation resulted in a small imaginary frequency of 5.3*i* cm^−1^, which could also arise due to accuracy limitations in the numerical integration of the DFT exchange‐correlation functional. Following this mode would lead to space group *Pc*, but the energy is lowered only by 0.1 kJ mol^−1^ per formula unit compared to *P*2_1_/*c*. The intermolecular interactions in the crystal structure of LT‐BrF_5_ were underestimated without the empirical dispersion correction, resulting in poor prediction of the Br−F bond lengths, F−Br−F angles, the crystal structure and the lattice parameters. Without the dispersion correction, the Br⋅⋅⋅F_eq_ contacts, responsible for the formation of one‐dimensional strands, are with 3.106 Å significantly longer than in the experimentally determined crystal structure with 2.881(3) Å, and differ only slightly from the Br−F_eq_ distances between the different strands, so that the motive of isolated strands was no longer discernible. The errors in lattice parameters with and without dispersion correction are shown in Table 3. Dispersion correction reduced the error in the lattice parameters to 1–2 %, and the strand motive was correctly described with the shortest intermolecular Br⋅⋅⋅F_eq_ distance being 2.946 Å compared to 2.881(3) Å in LT‐BrF_5_.

**Table 3:** Comparison of the calculated and observed lattice parameters of LT‐ and HT‐BrF_5_.^[a]^

Structure	*a* [Å]	*b* [Å]	*c* [Å]	*β* [°]	*V* [Å^3^]	Error [%]				
						*a*	*b*	*c*	*β*	*V*
LT‐BrF_5_ observed (100 K)	6.336	7.217	7.780	94.3	355					
LT‐BrF_5_ DFT‐PBE0	8.378	6.290	8.720	100.3	452	32	−13	12	6	27
LT‐BrF_5_ DFT‐PBE0‐D3	6.385	7.272	7.845	97.0	362	1	1	1	3	2
HT‐BrF_5_ observed (150 K)	7.829	6.386	7.236	–	362					
HT‐BrF_5_ DFT‐PBE0	8.154	7.101	7.352	–	426	4	11	2	–	18
HT‐BrF_5_ DFT‐PBE0‐D3	8.091	6.465	7.058	–	369	3	1	−2	–	2

[a] For HT‐BrF_5_, quantum‐chemical calculations with DFT‐PBE0‐D3 were also performed and LT‐BrF_5_ was determined to be 1.7 kJ mol^−1^ per formula unit lower in energy compared to HT‐BrF_5_ (calculations at 0 K). The structural motive is also well described by the calculation with errors between 0.9 and 1.4 % for the Br−F bond lengths and an error of 2.50 % for the intermolecular Br⋅⋅⋅F distance.

The energetics of the two polymorphs of BrF_5_ were further compared with the previously reported *Cmc*2_1_ structure^[13]^ and a hypothetical modification in the dimeric structure of AuF_5_.^[28]^ The previously reported *Cmc*2_1_ structure showed two imaginary frequencies of 33*i* and 32*i* cm^−1^, and was 2.9 kJ mol^−1^ per formula unit higher in energy compared to LT‐BrF_5_. For a hypothetical dimeric AuF_5_ structure (Au_2_F_10_), imaginary frequencies of over 300*i* cm^−1^ were present and the structure was 76 kJ mol^−1^ per formula unit higher in energy compared to LT‐BrF_5_, indicating that BrF_5_ is energetically very unlikely to adopt a dimeric molecular structure like AuF_5_. All optimized crystal structures are given in CIF format in the Supporting Information.

The optimized crystal structures and their energetics obtained from the quantum‐chemical calculations agree with the experimental results when a dispersion‐corrected DFT method is used. DFT calculations show that the LT‐ as well as the HT‐BrF_5_ crystal structures are energetically favored over the previously reported structural model and the phase transition from HT‐ to LT‐BrF_5_ could be followed by studying the imaginary vibrational modes obtained for HT‐BrF_5_.

### Reactions of BrF_5_ with *A*F (*A*=K, Rb)

By the reaction of BrF_5_ with the alkali metal fluorides *A*F (*A*=K, Rb), the corresponding alkali metal hexafluoridobromates(V), *A*[BrF_6_], are formed [Eq. 3].[^31^, ^32^]

(3) $$\mathrm{BrF}{}_{5}+AF\to A\left[\mathrm{BrF}{}_{6}\right]\;\;\left(A=K,\;\mathrm{Rb}\right)$$

While the crystal structure of Cs[BrF_6_] is already known,^[33]^ the crystal structures of K[BrF_6_] and Rb[BrF_6_] have not been reported. Suitable crystals of these compounds were obtained by slow cooling of a saturated solution of the respective alkali metal fluoride in BrF_5_ warmed to about 373 K in an air bath.

### Crystal structures of the compounds *A*[BrF_6_] (*A*=K, Rb)

**Figure 10:**
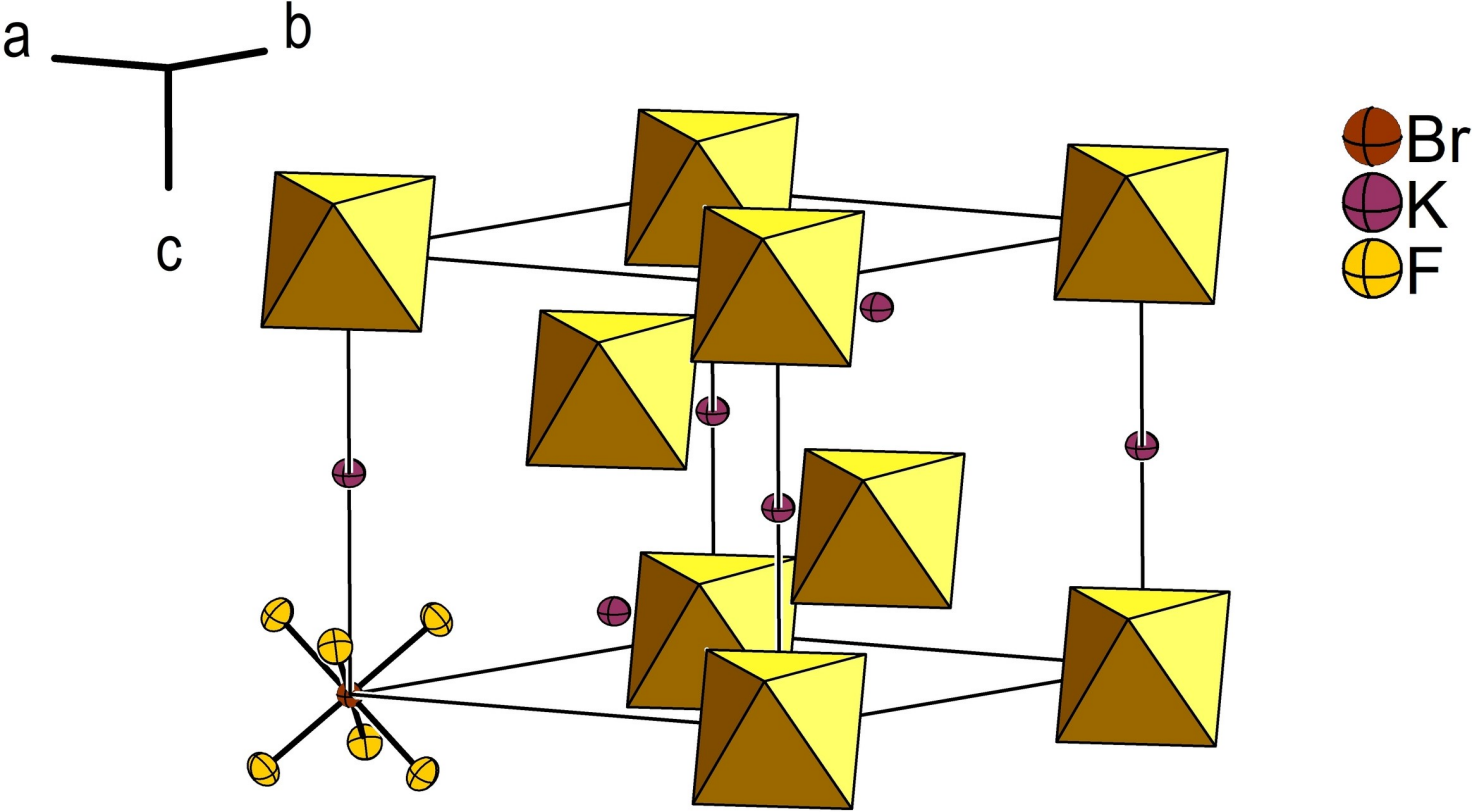
Crystal structure of K[BrF_6_]. The [BrF_6_]^−^ anions are shown as polyhedra, with the exception of the anion bottom left. Displacement ellipsoids are shown at the 70 % probability level at 100 K.

K[BrF_6_] and Rb[BrF_6_] crystallize isotypic to Cs[BrF_6_]^[33]^ in the K[AsF_6_] structure type in the trigonal crystal system, space group *R* $\bar{3}$ (no. 148, *hR*24), with the lattice parameters *a*=7.4450(14), *c*=7.287(2) Å, *V*=349.80(17) Å^3^, *Z=*3, at *T*=100 K for K[BrF_6_] and *a*=7.576(3), *c*=7.568(5) Å, *V=*376.2(4) Å^3^, *Z=*3, at *T*=100 K for Rb[BrF_6_]. The compounds have already been studied by Bougon and co‐workers using powder X‐ray diffraction methods,^[32]^ and two possible space groups *R* $\bar{3}$ and *R* $\bar{3}$ *m* have been proposed. Accordingly, two possible structure types were considered, the K[AsF_6_] type in *R* $\bar{3}$ and the Ba[SiF_6_] type in *R* $\bar{3}$ *m*. We can clearly exclude space group *R* $\bar{3}$ *m* as a refinement of the crystal structures in that space group leads to a striking elongation of the displacement ellipsoids of the fluorine atoms perpendicular to the *m* mirror plane. The crystal structure of the compounds in space group *R* $\bar{3}$ is shown in Figure 10 using K[BrF_6_] as an example. Details of the structure solutions and refinements are given in Table 4 and the atomic coordinates and isotropic displacement parameters are given in Table 5.

**Table 4:** Selected crystallographic data and details of the structure determination of K[BrF_6_] and Rb[BrF_6_].

Formula	K[BrF_6_]	Rb[BrF_6_]
Molar mass [g ⋅ mol^−1^]	233.01	279.38
Space group (no.)	*R* $\bar{3}$ (148)	*R* $\bar{3}$ (148)
*a* [Å]	7.4450(14)	7.576(3)
*c* [Å]	7.287(2)	7.568(5)
*V* [Å^3^]	349.80(17)	376.2(4)
*Z*	3	3
Pearson symbol	*hR*24	*hR*24
*ρ*_*calc*._ [g ⋅ cm^−3^]	3.318	3.700
*μ* [mm^−1^]	9.742	17.870
Color	colorless	colorless
Crystal morphology	needle	needle
Crystal size [mm^3^]	0.29×0.22×0.15	0.2×0.08×0.02
*T* [K]	100	100
*λ* [Å] (Mo_Kα_)	0.71073	0.71073
No. of reflections	267	1195
*θ* range [°]	4.220 to 30.423	4.111 to 28.942
Range of Miller indices	−10≤*h*≤8,	−10≤*h*≤10,
	−4≤*k*≤10,	−10≤*k*≤10
	0≤*l*≤10	−10≤*l*≤10
Absorption correction	numerical	numerical
*T*_max_, *T*_min_	0.3988, 0.1359	0.1811, 0.0551
*R*_int_, *R*_*σ*_	0.0318, 0.0298	0.0125, 0.0106
Completeness of the data set	1	1
No. of unique reflections	237	225
No. of parameters	14	14
No. of restraints	0	0
No. of constraints	0	0
*S* (all data)	1.016	0.971
*R*(*F*) (*I*≥2*σ*(*I*), all data)	0.0180, 0.0189	0.0173, 0.0254
*wR*(*F* ^*2*^) (*I*≥2*σ*(*I*), all data)	0.0420, 0.0422	0.0384, 0.0403
Largest diff. peak/hole [e Å^−3^]	0.56/−0.31	0.38/−0.31

**Table 5:** Positions, site symmetries, atomic coordinates and equivalent isotropic displacement parameters *U_iso_* for K[BrF_6_] and Rb[BrF_6_].

Compound	Atom	Position	*x*	*y*	*z*	*U_iso_* [Å^2^]
K[BrF_6_]	Br	3*a* ($\bar{3}$ .)	0	0	0	0.01213(15)
	K	3*b* ($\bar{3}$ .)	0	0	1/2	0.0171(2)
	F	18 *f* (1)	0.1579(2)	0.22651(18)	0.15221(16)	0.0191(3)
Rb[BrF_6_]	Br	3*a* ($\bar{3}$ .)	0	0	0	0.01903(16)
	Rb	3*b* ($\bar{3}$ .)	0	0	1/2	0.01613(17)
	F	18 *f* (1)	0.1590(2)	0.2219(2)	0.1457(2)	0.0237(3)

The bromine atoms reside on position 3*a* ($\bar{3}$ . ) and are coordinated octahedron‐like by six symmetry‐equivalent fluorine atoms (18 *f*, 1) forming the [BrF_6_]^−^ anion with point group symmetry *S*_6_. As already discussed by Seppelt and co‐workers for the crystal structure of Cs[BrF_6_], the lone pair of the Br atom within the [BrF_6_]^−^ anion seems to show no stereochemical activity.^[33]^ For a discussion on the lone pair, see below. The alkali metal cations (3*b*, $\bar{3}$ .) are 12‐fold coordinated by fluorine atoms in a cuboctahedron‐like environment.

Seppelt and co‐workers observed a Br−F bond length within the [BrF_6_]^−^ anions of Cs[BrF_6_] of 1.854(1) Å by X‐ray diffraction on single crystals and 1.847(1) Å by powder neutron diffraction.^[33]^ We found 1.8637(12) Å within the crystal structure of K[BrF_6_] and 1.8623(15) Å for Rb[BrF_6_], respectively, and quantum chemically calculated a Br−F bond length of 1.868 Å for the [BrF_6_]^−^ anion in the gas phase and of both circa 1.864 Å for the calculated crystal structures of K[BrF_6_] and Rb[BrF_6_]. While the observed Br−F bond lengths agree within the tripled standard uncertainties for the Rb and Cs compounds, the Br−F bond length is slightly longer in the determined crystal structure of the K compound. The observed bond lengths agree with those quantum chemically calculated for the solids and the Br−F bonds are longest in the gas‐phase [BrF_6_]^−^ anion, as may be expected.

Observed selected F−Br−F bond angles are 91.79(6)° within the potassium, 91.47(8)° within the rubidium, and 90.8(1)° within the cesium compound (91.3° by powder neutron diffraction). While the calculated gas‐phase [BrF_6_]^−^ anion shows ideal octahedral symmetry, the selected F−Br−F bond angles within the [BrF_6_]^−^ anions of the quantum chemically calculated crystal structures of K[BrF_6_] and Rb[BrF_6_] are 91.77° and 91.30°, respectively. Therefore, observed and quantum chemically calculated values agree. Based on these findings we conclude that the influence of the K^+^ cations on the molecular structure of the [BrF_6_]^−^ anions is seemingly the strongest and weakest for the Cs^+^ cations, as might have been expected on the basis of Pearson's concept of hard and soft acids and bases.^[34]^

### Raman spectroscopic investigation of the compounds *A*[BrF_6_] (*A*=K, Rb)

**Figure 11:**
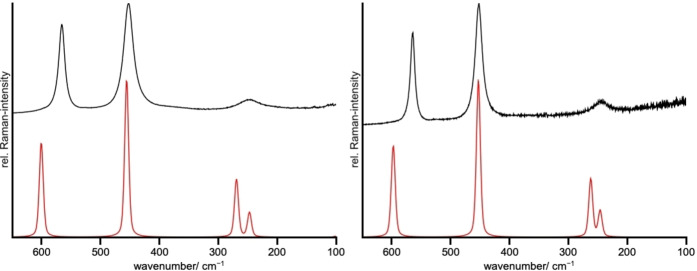
Raman spectra of K[BrF_6_] (left) and Rb[BrF_6_] (right) at room temperature recorded with an excitation laser wavelength of 532 nm, calculated spectra shown in red. No additional bands were present in the region above 600 cm^−1^ and up to 4000 cm^−1^.

The bulk phases of the samples were analyzed by Raman spectroscopy. For both, K[BrF_6_] and Rb[BrF_6_], three bands were observed corresponding to the Raman active vibrations of the octahedron‐like [BrF_6_]^−^ anion. The Raman spectra are shown in Figure 11 and the band assignments are given in Table 6.

**Table 6:** Band assignment for the Raman spectra of K[BrF_6_] and Rb[BrF_6_]. Raman inactive vibrations are not shown. Frequencies are given in cm^−1^.

Assignment	K[BrF_6_]		Rb[BrF_6_]		Description
	exp.	calc.	exp.	calc.	
*A_g_*	566	600	564	597	symmetric stretching vibration ν_s_. (in‐phase)
*E_g_*	452	456	452	452	symmetric stretching vibration ν_s_. (out‐of‐phase)
*E_g_*	248	269	244	261	symmetric in‐plane bending *δ_s_*
*A_g_*	248	246	244	245	symmetric out‐of‐plane bending *π_s_* (out‐of‐phase)

The observed Raman bands agree with the ones reported in the literature^[32]^ where the assignment of bands had been carried out using *O_h_* symmetry, while we used the crystallographic site symmetry of *S*_6_. We also see an agreement with the calculated Raman spectra (Figure 11). The calculated bands at 246 cm^−1^ for K[BrF_6_] and at 245 cm^−1^ for Rb[BrF_6_] are likely not observed due to their small intensities in comparison to the other bands or due to band overlap.

### Lone pair effects

**Scheme 2:**
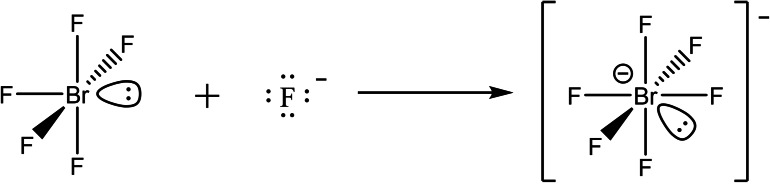
Lewis acid‐base reaction of BrF_5_ and F^−^ to [BrF_6_]^−^. The reaction might be counter‐intuitive when considering the lone pair on the Br atom of BrF_5_, which should repel the F^−^ anion. Also, no octahedron‐like structure might be expected for the [BrF_6_]^−^ anion because of the presumed orientation and shape of the lone pair.

Canonical molecular orbitals from quantum‐chemical calculations are typically rather difficult to interpret when chemical bonds between two or a few atoms are considered. Many chemists like to think of localized chemical bonds, draw molecular structures with Lewis formulas, and predict the shapes of molecules with simple models such as the VSEPR theory.[^35^, ^36^, ^37^] These however may bias judgment: For example, the action of BrF_5_ as a Lewis acid might be unexpected as one may think that the free valence electron pair, the lone pair, on the Br atom of the BrF_5_ molecule should repel an incoming F^−^ anion and therefore no [BrF_6_]^−^ anion would form based on this over‐simplified view. However, BrF_5_ acts as a Lewis acid under formation of [BrF_6_]^−^ anions according to Scheme 2, for examples see above and the literature.[^31^, ^33^, ^38^] Previous gas‐phase DFT−B3LYP calculations suggest the reaction in Scheme 2 to be exoenergetic by over 300 kJ mol^−1^.^[39]^ We obtained a gas‐phase reaction energy of −299 kJ mol^−1^ at the CCSD(T)/cc‐pVTZ level of theory. Furthermore, we estimated the Fluoride Ion Affinity[^40^, ^41^, ^42^, ^43^, ^44^] (FIA) of BrF_5_ to be 276 kJ mol^−1^ at the same level of theory (COF_2_ as reference system). With that it is comparable to PCl_3_ and PBr_3_, it is stronger than AsF_3_ and SF_4_, weaker than SbF_3_, SiF_4_, BF_3_ and much weaker than AsF_5_, SbF_5_ or Sb(OTf)_5_.^[43]^

**Figure 12:**
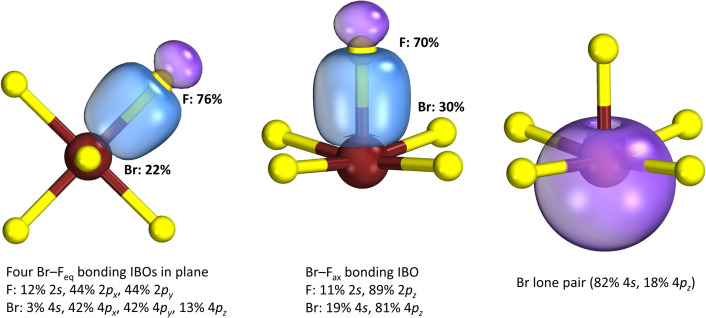
Intrinsic bond orbitals showing the Br−F bonds and the Br lone pair of the BrF_5_ molecule. Percentages next to the IBOs indicate the contribution of each atom to that IBO. The larger a percentage, the more polarized the covalent bond. If the summation does not add up to 100 %, then other atoms contribute less than 2 % to the IBO. The percentages in the labels show the contributions of atomic orbitals to each IBO. F atoms are in yellow, Br atoms in reddish‐brown. IBO isosurfaces are drawn so that 80 % of the density is enclosed within them.

In order to describe the chemical bonds of the BrF_5_ and [BrF_6_]^−^ molecules, population analyses were carried out at the DFT‐PBE0/TZVP level of theory by using intrinsic atomic orbitals (IAOs) and the bonding was analyzed with the aid of IBOs.[^45^, ^46^] For the gas‐phase BrF_5_ molecule, a local minimum is obtained in point group *C*_4*v*_, as may be expected. The Br atom shows a positive partial charge of +2.36 e^−^, while the four F_eq_ atoms show negative partial charges of −0.49 e^−^ and the apical fluorine atom F_ap_ a smaller charge of −0.40 e^−^. The high positive partial charge on the Br atom is surely also a reason why BrF_5_ reacts with a fluoride anion. Figure 12 shows selected IBOs for the BrF_5_ molecule. As expected, the Br−F bonds are strongly polarized covalent. The Br−F_eq_ bonds are essentially p‐like with a small 4 s contribution of the Br atom of 3 %. The Br−F_ap_ bond is slightly less polarized in comparison and the Br 4 s orbital contributes with 19 % to the bond. The shape of the lone pair on the Br atom is essentially s orbital‐like with only 18 % contribution from the 4p_z_ orbital.

**Figure 13:**
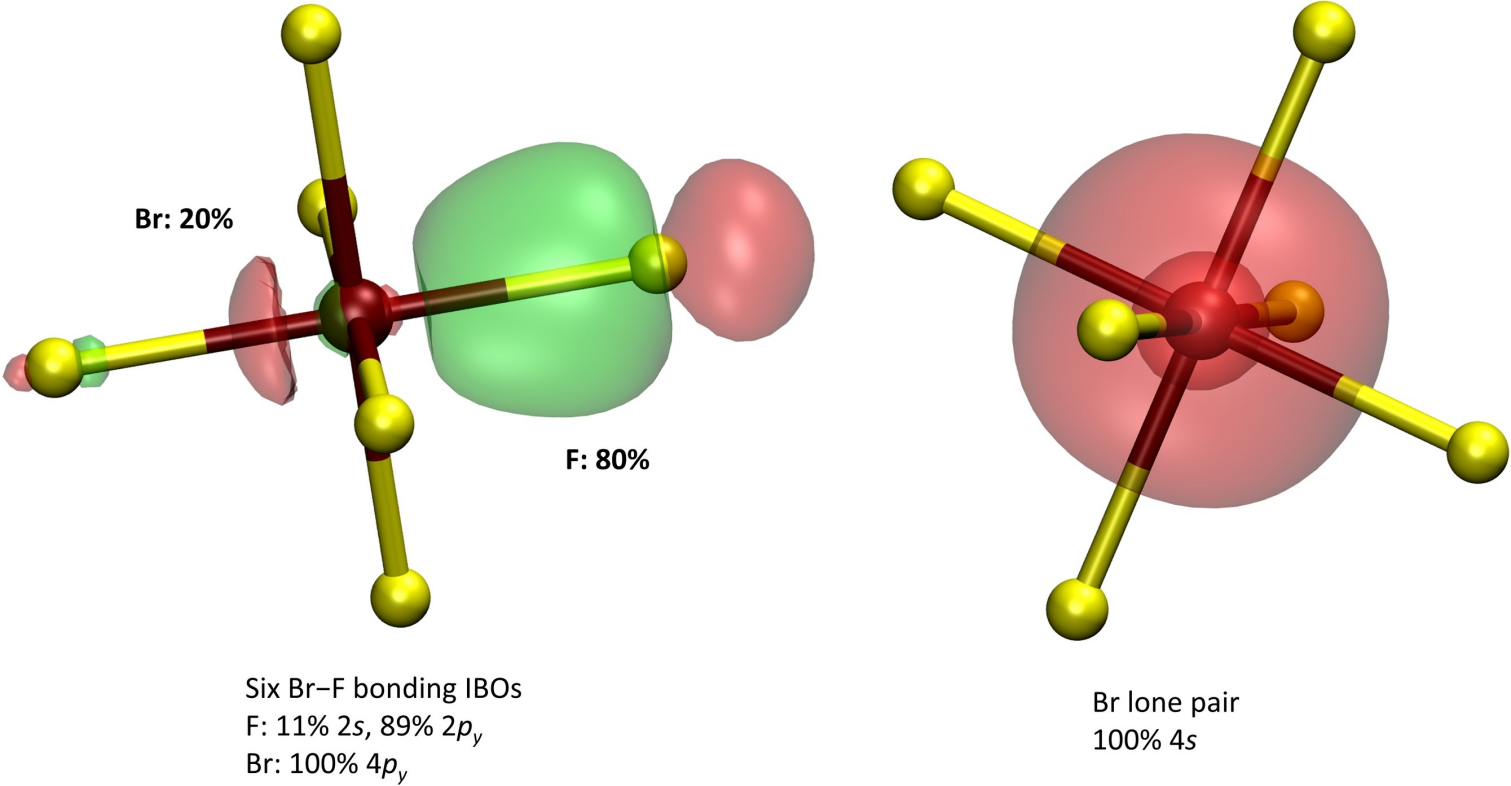
Intrinsic bond orbitals showing the Br−F bonds and the Br lone pair of the [BrF_6_]^−^ anion. Percentages next to the IBOs indicate the contribution of each atom to that IBO. The larger a percentage, the more polarized the covalent bond. The percentages in the labels show the contributions of atomic orbitals to each IBO (for Br−F bonding IBOs, the example orbital comprises p_*y*_ orbitals). F atoms are in yellow, Br atoms in reddish‐brown. IBO isosurfaces are drawn so that 80 % of the density is enclosed within them.

Quantum‐chemical calculations lead to a true local minimum for the [BrF_6_]^−^ anion in *O_h_* symmetry, that is, an “ideal” octahedron is obtained with no signs of stereochemical activity of the Br lone pair. The Br atom shows a positive partial charge of +2.32 e^−^ which is by 0.04 e^−^ lower compared to the Br atom within the BrF_5_ molecule. The F atoms show negative partial charges of −0.55 e^−^. Figure 13 shows selected IBOs of the [BrF_6_]^−^ anion.

The Br−F bonds in the [BrF_6_]^−^ anion become even more polarized compared to the BrF_5_ molecule and the Br atom no longer contributes with its 4 s orbital to the Br−F bonds but only with its p orbitals. The lone pair of the Br atom is completely 4 s‐like and therefore shows the absence of any stereochemical activity.

**Figure 14:**
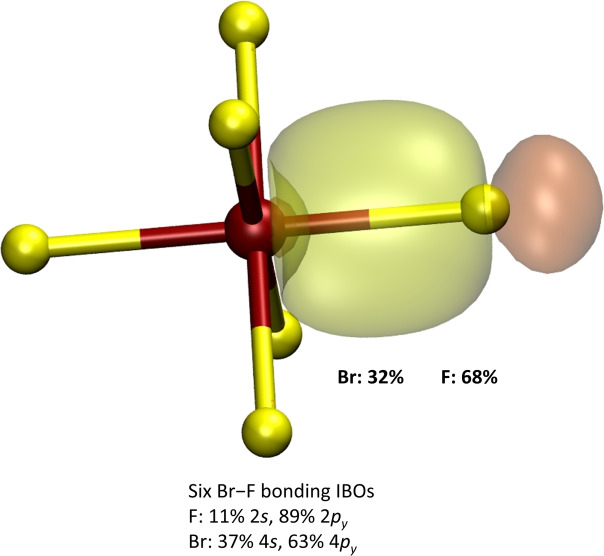
Intrinsic bond orbitals showing the Br−F bonds of the [BrF_6_]^+^ cation. Percentages next to the IBO indicate the contribution of each atom to the IBO. The larger a percentage, the more polarized the covalent bond. The percentages in the label show the contributions of atomic orbitals to the shown example IBO. F atoms are in yellow, Br atoms in reddish‐brown. IBO isosurfaces are drawn so that 80 % of the density is enclosed within them.

We calculated the [BrF_6_]^+^ cation, where there is no Br lone pair, for comparison and found a true local minimum for *O_h_* symmetry, as expected from the literature.[^7^, ^47^] The calculated Br−F bond length is 1.67 Å which agrees with estimated 1.69 Å^[33]^ based on its reported force constants.^[47]^ Its IBOs of the Br−F bonds are shown in Figure 14.

In the [BrF_6_]^+^ cation the Br−F bonds are less polarized in comparison to [BrF_6_]^−^ and also in comparison to the BrF_5_ molecule. The contribution of the Br 4 s orbital to the Br−F bond has increased to 37 %.

For the species discussed here, [BrF_6_]^+^, BrF_5_, and [BrF_6_]^−^, the F atoms always contribute more or less the same to the Br−F bonds, circa 11 % with their 2 s and about 89 % with the respective 2p orbital. The 4 s orbital contribution to the Br−F bonds goes from 37 % in the [BrF_6_]^+^ cation down to 20 % for Br−F_ap_ and 3 % for Br−F_eq_ in the BrF_5_ molecule, and reaches 0 % in [BrF_6_]^−^. The 4p orbital contributions follow the inverted trend, the Br−F bonds are strongest p‐like in [BrF_6_]^−^ and least in [BrF_6_]^+^.

The stereochemical inactivity of the lone pair of the Br atom within the [BrF_6_]^−^ anion had been discussed previously by different researchers. Christe and co‐workers reasoned well‐founded that “in a rigid molecule the space requirement of a sterically active free valence electron pair slightly exceeds that of a fluorine ligand”,^[48]^ and that therefore anions such as [IF_6_]^−^ are not octahedral as there is enough space around the I atom for the lone pair to become active because I atoms can adopt coordination numbers up to eight with F ligands, for example, in [IF_8_]^−^ anions. “In [BrF_6_]^−^ there is little or no room left for a seventh ligand and, therefore, the free valence electron pair should be sterically inactive” they concluded.^[38]^ Seppelt and co‐workers had the opinion that the 4 s electrons were particularly strongly bound to the Br atomic nucleus as the fully occupied 3d shell shields the nuclear charge incompletely. Therefore, the Br−F bonds would mainly show p character.^[33]^ Gillespie and co‐workers reasoned that the six F atoms around the small Br atom are close packed. Therefore, no room for the lone pair is left and it belongs to the Br core.[^36^, ^49^]

**Figure 15:**
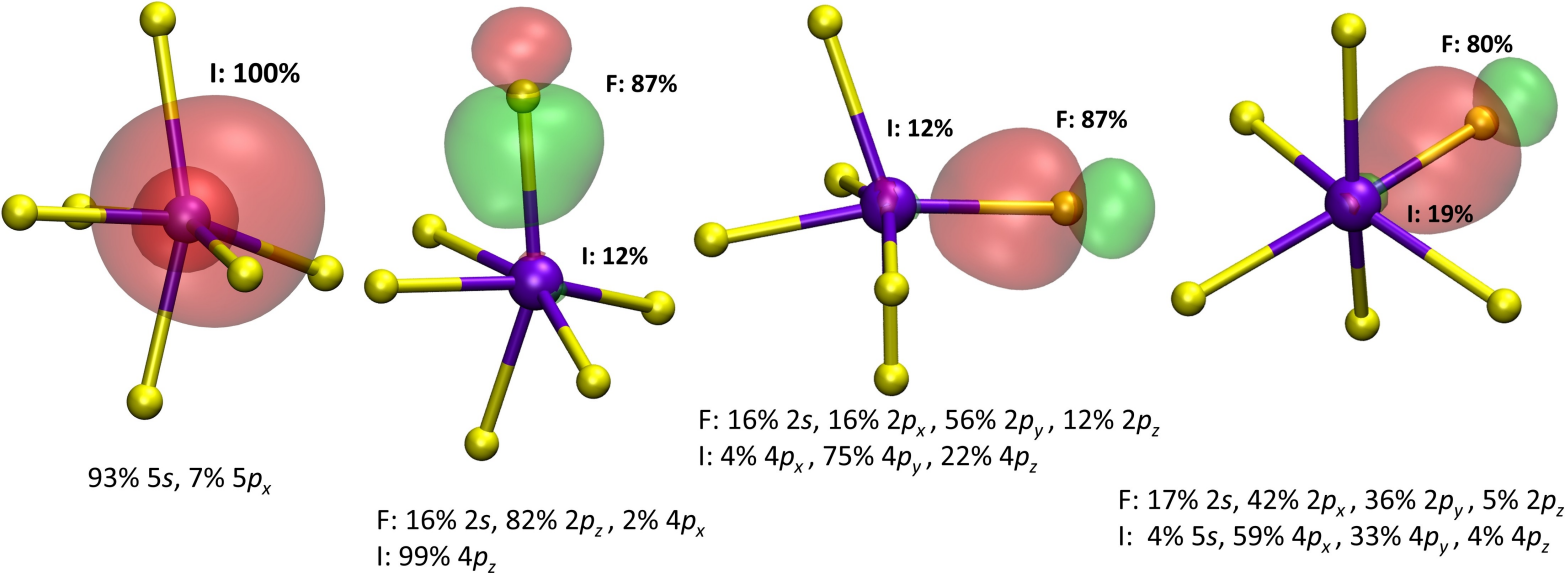
IBOs of the I lone pair and the I−F bonds of the [IF_6_]^−^ anion in its energy minimum in point group *C*_1_. Percentages next to the IBOs indicate the contribution of each atom to that IBO. The larger a percentage, the more polarized the covalent bond. The percentages in the labels show the contributions of atomic orbitals to each IBO. F atoms are in yellow, I atoms in violet. IBO isosurfaces are drawn so that 80 % of the density is enclosed within them.

A quantum‐chemical calculation of the [IF_6_]^−^ anion shows that its molecular structure is not a local minimum in point group *O_h_*. Following the imaginary mode of 39*i* cm^−1^ leads to molecular structures in point groups *C*_2*v*_ and *C_s_* that are also no local minima. Finally, a true local minimum in point group *C*_1_ is reached, and overall the energy lowered by 14 kJ mol^−1^ compared to the ideal *O_h_* symmetry. The IBOs of the I lone pair and the I−F bonds of the [IF_6_]^−^ anion in its energy minimum are shown in Figure 15.

The I lone pair is essentially 5 s‐orbital‐like with 93 % 5 s and 7 % 5p_*x*_ orbital contribution. It therefore shows some stereochemical activity in contrast to the Br lone pair of the [BrF_6_]^−^ anion. Both reasons for the stereochemical inactivity of the Br lone pair of the [BrF_6_]^−^ anion given by Seppelt or Christe are valid and in agreement with our quantum chemical findings.

## Conclusions

BrF_5_ was synthesized at room temperature by the photochemical reaction of BrF_3_ with F_2_. UV‐Vis spectra of BrF_5_ and F_2_ were recorded to identify the region of suitable wavelength, 300 to 400 nm, to photodissociate F_2_ but not the product BrF_5_. BrF_5_ can be obtained in yields above 90 % with respect to the starting material BrF_3_ and pure on the basis of NMR, IR and Raman spectroscopy. NMR spectra of BrF_5_ were taken at various temperatures. Quantum‐chemical calculations aided in the determination of the new low‐temperature modification and allowed the band assignment of the recorded IR and Raman spectra. By treating BrF_5_ with KF and RbF, crystals of the compounds K[BrF_6_] and Rb[BrF_6_], respectively, were obtained and their crystal structures determined. The chemical bonds and the lone pairs on the Br atoms within the molecules [BrF_6_]^+^, BrF_5_, and [BrF_6_]^−^ were investigated by using intrinsic bond orbitals. These show that the contribution of the 4 s orbitals of the Br atoms to the Br−F bonds decreases to zero in the octahedral [BrF_6_]^−^ anion where the Br lone pair is purely 4 s‐orbital‐like. In the [IF_6_]^−^ molecule, which is not octahedral, the 5p orbitals contribute to the I lone pair, making it stereochemically active.

## Experimental Section

**General**: All operations were performed on a Monel metal Schlenk line, which was passivated with fluorine and ClF_3_ at various temperatures and pressures before use. The alkali metal fluorides were purchased from *Merck*, purified according to literature procedures,^[50]^ and were stored in PTFE vials in an Ar‐filled glove box (MBraun).

Reaction vessels were made out of fluoropolymer (perfluoroalkoxy alkanes, PFA or perfluorinated ethylene propylene copolymer, FEP) and sealed with a bellows valve made out of Monel or stainless steel. The vessels were baked out in vacuum (∼10^−3^ mbar) at circa 393 K for several times and then filled up to a pressure of 4 bar with diluted F_2_ (F_2_/Ar 20 : 80, *v*/*v*) for 16 h in order to saturate the polymer with fluorine.

***CAUTION***! Fluorine, the halogen fluorides and fluoridobromates(V) must be handled with appropriate protective gear with ready access to proper emergency treatment procedures in the event of contact. The aforementioned are potent oxidative fluorinators that are only stable under the rigorously anhydrous conditions employed in the experimental procedures outlined in the Experimental Section. They react vigorously to explosively upon hydrolysis or contact with organic materials. The utmost precautions must be taken when disposing of these materials and their derivatives.

**Preparation of BrF_5_ in PFA vessels**: When only small amounts of BrF_5_ around 1 g are required, the reaction can be performed in a reaction vessel with a volume of 50 to 80 mL made out of PFA or FEP and equipped with a stainless‐steel valve.

For a typical synthesis 692 mg (5.08 mmol) BrF_3_ was loaded into the reaction vessel (*V*=74 mL) and diluted fluorine (F_2_/Ar 20 : 80, *v*/*v*, 3 bar, 1.79 mmol) was added. After irradiating the reaction mixture with a UV LED (Osram, LuxiGen‐UV395, 395 nm, 1380 mW) for 6 h, the reaction vessel was cooled to 77 K and all volatiles were pumped off. The vessel was then allowed to warm to room temperature and diluted fluorine (3 bar, 1.79 mmol) was added again. This process was repeated for three times. After the third addition of F_2_ a slight excess (5.37 mmol) with respect to Br was reached. The liquid completely decolorized upon irradiation, which can be attributed to the absence of BrF_3_. Bromine pentafluoride (792 mg, 4.55 mmol, 90 % with respect to the starting material BrF_3_) was obtained as a colorless liquid. As BrF_5_, like ClF_5_,^[5]^ tends to dissolve in the PFA/FEP vessel wall over time, the yield decreases with longer reaction times. Therefore, for larger batches requiring longer reaction times, vessels made of PFA/FEP should be avoided if possible.

**Figure 16:**
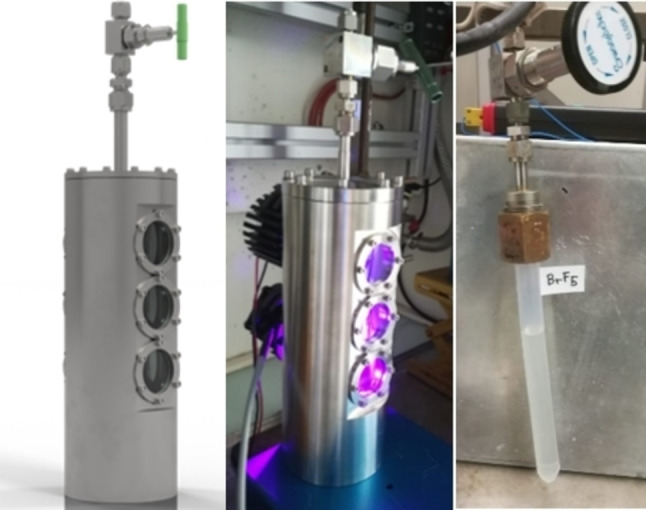
Rendered graphic of the stainless‐steel photoreactor (left) used for the synthesis of BrF_5_. Photographs of the reactor during the reaction (middle) and the PFA vessel containing the product (right). Photographs by Martin Möbs.

**Preparation of BrF_5_ in a stainless‐steel reactor**: For the preparation of larger amounts of circa 60 g BrF_5_ per batch, a stainless‐steel (1.4571) reactor (Figure 16) with sapphire windows was used. For a typical synthesis 48.46 g (354 mmol) BrF_3_ was loaded into the reactor (*V*=924 mL) and undiluted fluorine (1 bar, 42.7 mmol) was added. The reaction mixture was irradiated with a UV LED (Nichia, NVSU233B, 365 nm, 1450 mW) for 12 h during which the pressure dropped to circa 400 mbar. The F_2_ pressure was then again increased to 1 bar and the process was repeated until the reaction mixture completely decolorized after 9.5 d of irradiation. Bromine pentafluoride (59.11 g, 338 mmol, 95 % with respect to BrF_3_) was obtained as a colorless liquid.

**Preparation of** ***A*** **[BrF_6_] (*A*=K, Rb)**: A PFA reaction vessel treated as described above was charged in a glove box with 40 mg of the respective dry alkali metal fluoride. The reaction vessel was then cooled to 77 K and an excess of BrF_5_ (0.1 mL, 246 mg, 1.4 mmol) was condensed. The reaction mixture was then allowed to warm to room temperature and diluted fluorine was added to a pressure of 3 bar to counteract diffusion of external moisture and air. The reaction mixture was heated to circa 373 K in a hot air bath for 1 h. By slowly cooling the mixture down to room temperature, the product was obtained in form of colorless, needle shaped crystals. After a few crystals were transferred from the BrF_5_ solution directly into the perfluorinated oil for the single‐crystal preparation, BrF_5_ was removed from the reaction vessel under reduced pressure and the remaining solid was heated to 373 K in vacuum for 1 h in order to release any adsorbed BrF_5_. We were not able to obtain the compounds phase‐pure this way, there seems to be still too much diffusion through the PFA vessels because the HF_2_^−^ salts were formed in varying amounts during the syntheses.

**NMR spectroscopy**: ^19^F NMR spectra were recorded using a Bruker Avance III HD 300 NMR spectrometer. CFCl_3_ was used as an external standard. A sample of neat BrF_5_ was distilled into a thoroughly baked out 3 mm diameter FEP tube and sealed under vacuum. The sample was then placed into a regular glass NMR tube (5 mm) and stored under argon at 237 K until assayed.

**Raman spectroscopy**: The Raman spectra were measured with a Monovista CRS+ confocal Raman microscope (Spectroscopy & Imaging GmbH) using a 532 nm solid‐state laser and either a 300 grooves/mm (low‐resolution mode, FWHM: <4.62 cm^−1^) or an 1800 grooves/mm (high‐resolution mode, FWHM: <0.368 cm^−1^) grating. Sample preparation of BrF_5_: A silica capillary was baked out under vacuum and flushed with diluted fluorine for several times. BrF_5_ was distilled at liquid nitrogen temperature into the capillary which was then flame‐sealed under vacuum. The capillary was allowed to warm to room temperature and was placed under the Raman microscope for data acquisition. Sample preparation of *A*[BrF_6_] (*A*=K, Rb): Samples were filled and sealed in quartz capillaries inside the glovebox and were then placed under the Raman microscope for data acquisition.

**Infrared spectroscopy**: The gas‐phase IR spectrum of BrF_5_ was recorded on a Bruker Tensor 37 FTIR with a resolution of 4 cm^−1^ using a passivated measuring cell manufactured from 316 L stainless steel, equipped with diamond or BaF_2_ windows. IR spectra of solids were recorded on a Bruker alpha FTIR spectrometer using the ATR Diamond module with a resolution of 4 cm^−1^. The spectrometer was located inside a glovebox (MBraun) under argon atmosphere. The spectra were processed with the OPUS software package.^[51]^

**Single‐crystal X‐ray diffraction**: A crystal of K[BrF_6_] or Rb[BrF_6_], respectively, was selected under pre‐dried perfluorinated oil (Fomblin YR‐1800) and mounted using a MiTeGen loop. Intensity data of a suitable crystal were recorded with an IPDS 2 diffractometer (Stoe & Cie). The diffractometer was operated with Mo_Kα_ radiation (0.71073 Å, graphite monochromator) and equipped with an image plate detector. In case of K[BrF_6_], split reflections were observed for several crystals, which indicated non‐merohedral twinning. Evaluation, integration and reduction of the diffraction data was carried out using the X‐Area software suite.^[52]^ A numerical absorption correction was applied with the modules X‐Shape and X‐Red32 of the X‐Area software suite. The structures were solved with dual‐space methods (SHELXT‐2014/5) and refined against *F* ^2^ (SHELXL‐2018/3).[^53^, ^54^]

For the structure solution of K[BrF_6_] only the non‐overlapping reflections of the major twin component were used. The data were initially refined with the HKLF5 format option in SHELXL‐2018/3 with all reflections (overlapping reflections and non‐overlapping reflections of three twin components).^[54]^ The data were then processed with the HKLF5Tools^[55]^ program: the non‐overlapping reflections of the weaker diffracting twin components were removed, the non‐overlapping reflections of the major twin component were merged in point group $\bar{3}$ and the overlapping reflections were merged in point group 1. The final refinement was carried out against the detwinned dataset (created by SHELXL with the LIST 8 option as an FCF file and converted to an HKL file with HKLF5Tools).

For the determination of the crystal structure of BrF_5_, a sample of the latter was prepared in a 0.3 mm quartz capillary as described for its Raman spectroscopy and mounted on a D8 Quest diffractometer (Bruker). The diffractometer was operated with monochromatized Mo−Kα radiation (0.71073 Å, multi layered optics) and equipped with a PHOTON 100 CMOS detector. By cooling the sample to 200 K on the diffractometer, a polycrystalline material was obtained, as shown by the diffraction pattern in which Debye‐Scherrer rings are indicated. By repeated zone melting of the sample in the capillary with the help of a copper wire, a single crystal suitable for X‐ray structure determination was obtained. Full datasets of the crystal were measured at 200, 180, 150 and 100 K respectively. Evaluation, integration, and reduction of the diffraction data was carried out within the APEX3 software suite.^[56]^ The data was corrected for absorption utilizing the multi‐scan method of SADABS^[57]^ within the APEX3 software suite, the structure was solved with dual‐space methods (SHELXT‐2014/5) and refined against *F* ^2^ (SHELXL‐2018/3).[^53^, ^54^] When the crystal was cooled below about 130 K, split reflections were observed in the diffraction pattern, indicating that the crystal was shattered by a phase transition. In consequence, the diffraction data recorded at 100 K is not from a single crystal but contains reflections from multiple crystallites. Using the CELL_NOW^[58]^ indexing algorithm more than five domains with identical lattice parameters were found, of which only the two strongest were used for further processing. Only the non‐overlapping reflections of the major twin component were used for the structure solution. The structure was solved with dual‐space methods (SHELXT).^[53]^ The data were initially refined with the HKLF5 format option in SHELXL with all reflections (overlapping reflections and non‐overlapping reflections of the three twin components).^[54]^ The data were then processed with the HKLF5Tools^[55]^ program: the non‐overlapping reflections of the weaker diffracting twin components were removed, the non‐overlapping reflections of the major twin component were merged in point group 2/*m* and the overlapping reflections were merged in point group 1. The final refinement was carried out against the detwinned dataset (created by SHELXL with the LIST 8 option as a FCF file and converted to a HKL file with HKLF5Tools).

In the datasets recorded at 150, 180 and 200 K, several additional weak reflections occur at half the diffraction angle of particularly strong reflections. This can be attributed to the minor lambda‐half fraction of the primary X‐ray beam that is not filtered out by the monochromator and gets diffracted due to the big crystal size with significant intensity. As no subsequent correction was made, the goodness of fit values are somewhat higher than might be expected.

All atoms were refined with anisotropic displacement parameters. Representations of the crystal structures were created with the Diamond software.^[59]^

Deposition Numbers 2193268 (for K[BrF_6_]), 2193269 (for HT‐BrF_5_ at 200 K), 2193270 (for HT‐BrF_5_ at 150 K), 2193271 (for LT‐BrF_5_), 2193272 (for HT‐BrF_5_ at 180 K), 2193273 (for Rb[BrF_6_]) contain the supplementary crystallographic data for this paper. These data are provided free of charge by the joint Cambridge Crystallographic Data Centre and Fachinformationszentrum Karlsruhe Access Structures service.

**Powder X‐ray diffraction**: A sample of BrF_5_ was prepared in a 0.3 mm quartz capillary as described for Raman spectroscopy and single‐crystal X‐ray diffraction. The powder X‐ray pattern was recorded with a StadiMP diffractometer (Stoe & Cie) in Debye‐Scherrer geometry. The diffractometer was operated with Cu${}_{K\alpha {}_{1}}$ radiation (1.5406 Å, germanium monochromator) and equipped with a MYTHEN 1 K detector. The sample was cooled to 180 K using a capillary cooling system (Osford Cryosystems). Diffraction data of the resulting polycrystalline material were collected in eight subsequent runs in a range of 15 to 71 °2*θ* with an irradiation time of 80 s per 1°. Due to ice formation on the surface of the capillary during the measurement only the first tree runs were used for the refinement.

**Figure 17:**
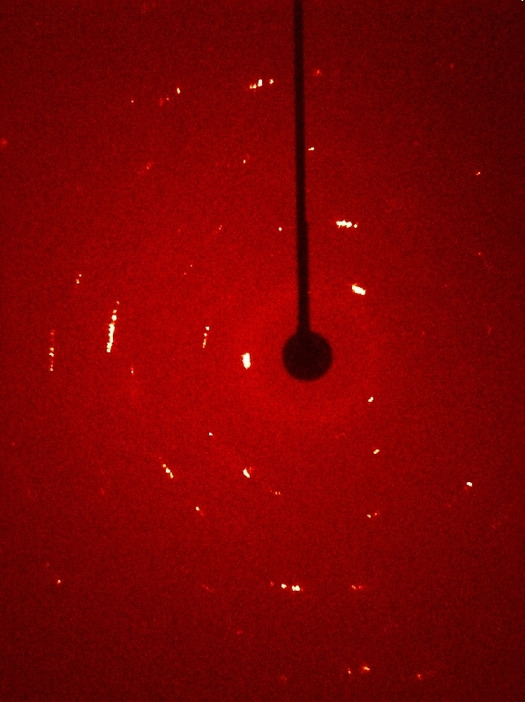
X‐ray diffraction image of a polycrystalline sample of BrF_5_ obtained by cooling the liquid to 180 K.

Rietveld refinements^[60]^ were performed using the TOPAS‐Academic software (version 7).^[61]^ The structure model derived from the X‐ray structure analysis of the single crystal was used as the starting point for the refinement. A shifted Chebyshev polynomial with 12 terms was used to describe the background, the peak profiles were fitted with a modified Thompson‐Cox‐Hastings pseudo‐Voigt (“TCHZ”) function as implemented in TOPAS, and the zero offset was refined. To account for absorption, an intensity correction for cylindrical samples was applied as implemented in TOPAS. An eighth‐order spherical harmonics function was used to account for the preferred orientation of the crystallites. The strong preferred orientation of the sample can be explained by the fact that the liquid BrF_5_ was frozen directly inside of the capillary, making it impossible to grind the sample before measurement. Therefore, various different directions of crystal growth are to be expected. The same phenomenon was observed when trying to obtain single crystals of the compound. In this case, the preferred orientation was also clearly visible in the diffraction image (Figure 17).

The approach of Le Bail and Jouanneaux^[62]^ was used to accurately describe the peak half‐width and shape anisotropy effects, and the corresponding parameters could be freely refined. The final refinement cycles converged with free refinement of all background, profile, and lattice parameters, including the coordinates and isotropic displacement parameters of all atoms.

**Computational details**: We carried out anharmonic force field and ^19^F NMR chemical shift calculations on the gas‐phase BrF_5_ molecule with CFOUR v2.1[^63^, ^64^] program suite. CCSD(T) coupled cluster method was used in combination with correlation‐consistent polarized triple‐zeta basis sets. For geometry optimizations and anharmonic frequency calculations, we used triple‐valence correlation consistent basis set (cc‐pVTZ)[^65^, ^66^] The structure was fully optimized within the *C*_4*v*_ point group (wavefunction was obtained for the highest Abelian subgroup *C*_2*v*_). The geometry of CFCl_3_ was also optimized and it was used as the NMR reference species for ^19^F NMR chemical shifts. The ^19^F NMR chemical shift calculations were carried out at the optimized geometries with weighted core‐valence basis sets (cc‐pwCVTZ).[^67^, ^68^] Anharmonic vibrational spectra were calculated within the second‐order vibrational perturbation theory (VPT2).^[69]^ For evaluating the Fluoride Ion Affinity (FIA) of BrF_5_, we optimized [BrF_6_]^−^, COF_2_, and COF_3_^−^ at the CCSD(T)/cc‐pVTZ level of theory and used the COF_2_ reference system with an experimental FIA of 208.08 kJ mol^−1^ to estimate the FIA at an absolute scale.^[43]^ The Z‐matrices of the optimized molecular structures are included in the Supporting Information.

All solid‐state calculations were performed with the CRYSTAL17^[70]^ program suite. HT‐ and LT‐BrF_5_, Rb[BrF_6_], and K[BrF_6_] were investigated with hybrid density functional methods (DFT‐PBE0)[^71^, ^72^] combined with triple‐valence basis sets TZVP for Br^[73]^ and F,^[74]^ and a split‐valence basis set SVP for K^[75]^ and Rb^[76]^ (derived from molecular Karlsruhe basis sets^[77]^). In case of the molecular crystals HT‐ and LT‐BrF_5_, empirical D3 dispersion correction with zero damping[^78^, ^79^] was applied for the geometry optimizations and frequency calculations to take into account weak intermolecular interactions. Atomic positions and lattice parameters were fully optimized within the space group symmetry of each system. The reciprocal space was sampled with Monkhorst‐Pack‐type *k*‐point grids.^[80]^ The employed *k*‐point meshes are reported in Table S5. Tight truncation criteria (TOLINTEG 8, 8, 8, 8, 16) were applied for the evaluation of the bielectronic Coulomb and exchange series in all calculations. Default DFT integration grids and optimization convergence thresholds were used in all calculations. Harmonic vibrational frequencies and Raman intensities were calculated with the schemes implemented in CRYSTAL.[^81^, ^82^, ^83^] Raman intensities were calculated for a polycrystalline powder sample. For Rb[BrF_6_] and K[BrF_6_] the experimental setup of *T*=298.15 K and *λ*=532 nm was considered. For the simulation of the spectra a pseudo‐Voigt band profile (50 : 50 Lorentzian/Gaussian) with a FWHM of 8 cm^−1^ was used. Assignment of the vibrational bands was done with the visualization tool CRYSPLOT.^[84]^ The atomic positions and lattice parameters of the optimized structures are given in the Supporting Information.

## Conflict of interest

The authors declare no conflict of interest.

1

## Supporting information

As a service to our authors and readers, this journal provides supporting information supplied by the authors. Such materials are peer reviewed and may be re‐organized for online delivery, but are not copy‐edited or typeset. Technical support issues arising from supporting information (other than missing files) should be addressed to the authors.

Supporting Information

## Data Availability

The data that support the findings of this study are available in the supplementary material of this article.
