# Supplementary material for: Bromine Pentafluoride BrF5, the Formation of [BrF6]− Salts, and the Stereochemical (In)activity of the Bromine Lone Pairs
Source: Chemistry. 2022 Oct 31;28(72):e202202466. doi: 10.1002/chem.202202466 (PMC10092034; doi:10.1002/chem.202202466)
Supplement: Supplementary file 1 — Supporting Information [file CHEM-28-0-s001.pdf]

Chemistry–A European Journal

Supporting Information

Bromine Pentafluoride BrF_5, the Formation of $[BrF_6]^-$ Salts, and the Stereochemical (In)activity of the Bromine Lone Pairs

Martin Möbs, Tim Graubner, Kim Eklund, Antti J. Karttunen, and Florian Kraus*

Table of Contents

NMR spectroscopic studies on BrF_5 2

Vibrational spectroscopic studies on BrF_5 4

Quantum chemical calculations 9

Z-Matrices for CFOUR calculations 9

Employed *k*-point meshes 13

Optimized geometries of the BrF_5 crystal structures in CIF format 13

Optimized geometries of the $K[BrF_6]$ and $Rb[BrF_6]$ crystal structures in CIF format 18

Calculated Raman spectra of LT-BrF_5 and HT-BrF_5 20

Rietveld refinement details 21

Crystallographic considerations 22

Variable-temperature powder X-ray diffraction on BrF_5 23

Author Contributions 24

NMR spectroscopic studies on BrF_5

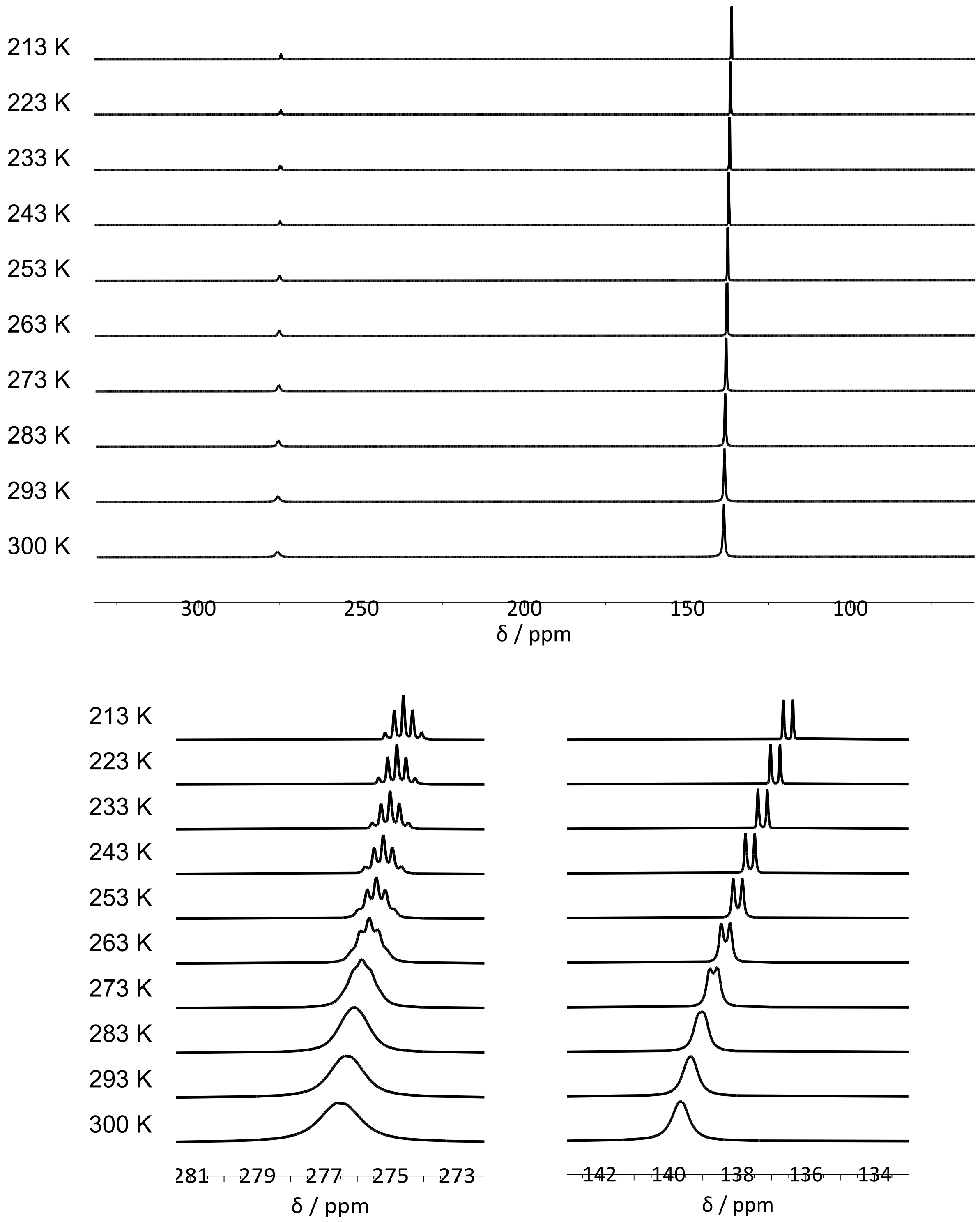

Figure S1: ^{19}F NMR spectra (above) and enlarged sections for the region of interest (below) of neat BrF_5 measured at temperatures from 300 to 213 K in a region from 0 to 400 ppm referenced to δ($CFCl_3$) = 0 ppm.

Table S1: NMR data of BrF_5 recorded at various temperatures in steps of 10 K from 213 to 300 K.

T / K	δ_{19F} / ppm ($CFCl_3$)	M	$J_{F\text{–}F}$ / Hz	Width / Hz
300	276.5 (F_{ax})	*s*		409.4
	139.7 (F_{eq})	*s*		165.3
293	276.3 (F_{ax})	*s*		342.5
	139.4 (F_{eq})	*s*		144.3
283	276.1 (F_{ax})	*s*		292.6
	139.1 (F_{eq})	*s*		133.0
273	275.9 (F_{ax})	*p*	67	108.6
	138.7 (F_{eq})	*d*		62.9
263	275.6 (F_{ax})	*p*	72	75.1
	138.3 (F_{eq})	*d*		43.9
253	275.4 (F_{ax})	*p*	75	51.6
	138.0 (F_{eq})	*d*		28.3
243	275.2 (F_{ax})	*p*	76	37.8
	137.6 (F_{eq})	*d*		20.4
233	275.0 (F_{ax})	*p*	76	30.1
	137.3 (F_{eq})	*d*		16.8
223	274.8 (F_{ax})	*p*	77	26.0
	136.9 (F_{eq})	*d*		15.7
213	274.61 (F_{ax})	*p*	77	24.2
	136.51 (F_{eq})	*d*		16.0

Vibrational spectroscopic studies on BrF_5

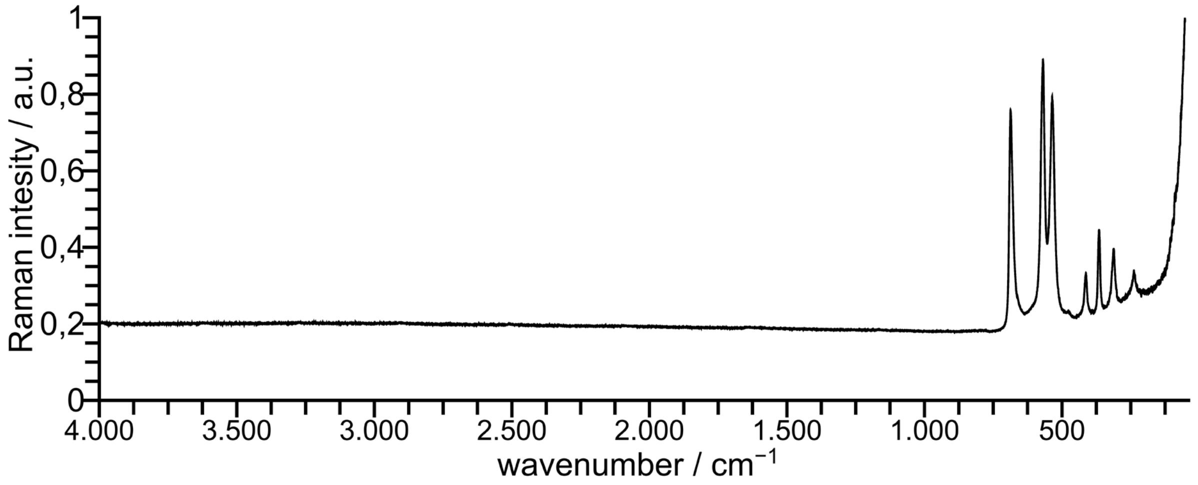

Figure S2: Raman spectrum of liquid BrF_5 recorded at room temperature in the range of 100 to 4000 cm^{-1} with an excitation laser wavelength of 532 nm.

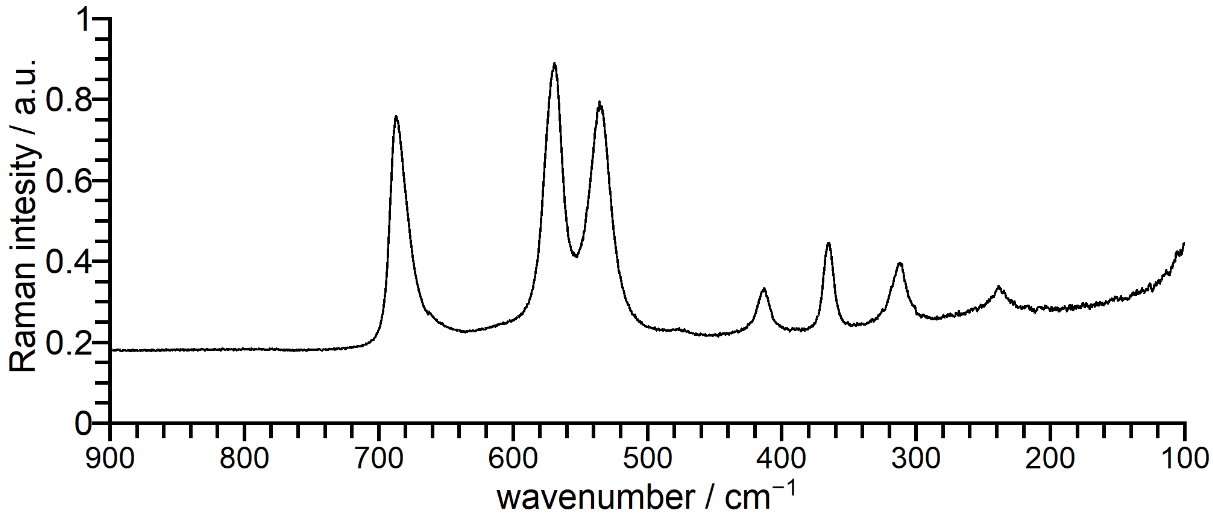

Figure S3: Enlarged section of the Raman spectrum of liquid BrF_5 at room temperature recorded with an excitation laser wavelength of 532 nm.

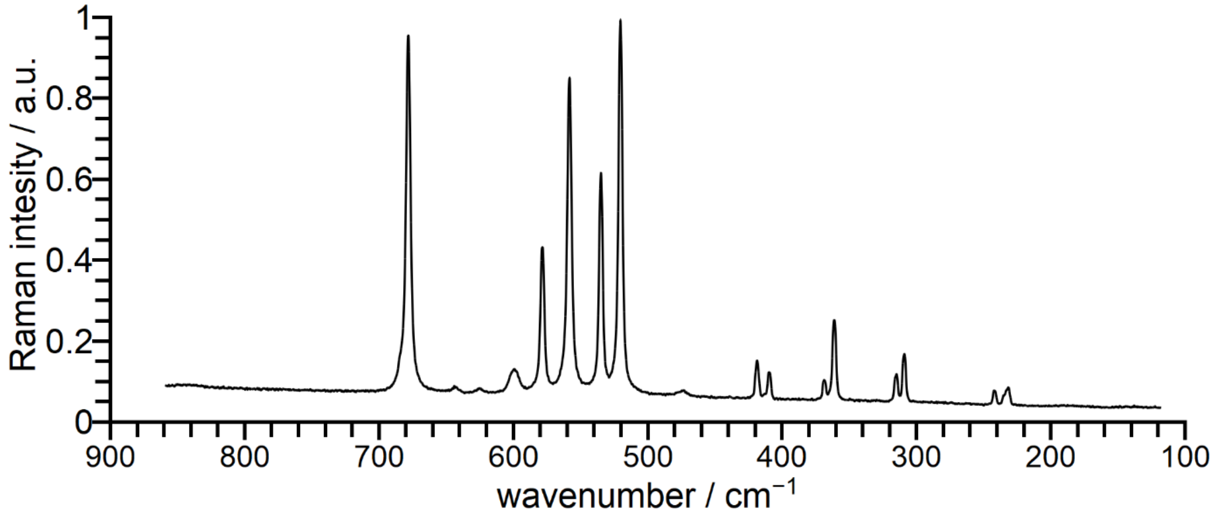

Figure S4: Raman spectrum of solid BrF_5 at recorded at 150 K with an excitation laser wavelength of 488 nm.

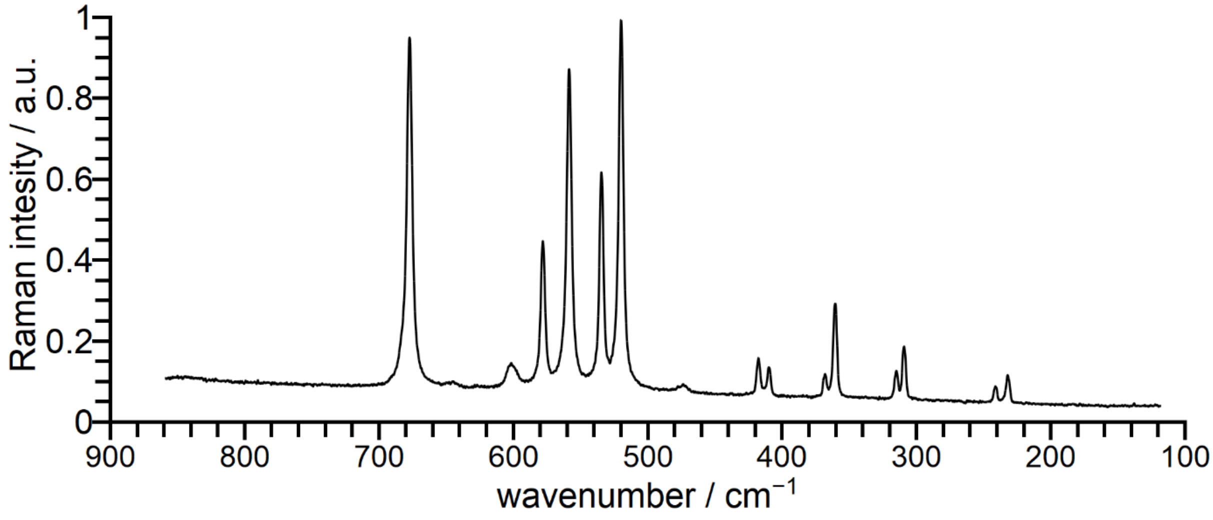

Figure S5: Raman spectrum of solid BrF_5 recorded at 100 K with an excitation laser wavelength of 488 nm.

Table S2: Calculated (DFT-PBE0-D3/TZVP) and observed Raman frequencies of solid BrF_5 (Pnma) at 100 and 150 K.

$\tilde{\nu}$ (calculated) / cm^{-1}	$\tilde{\nu}$ (measured) / cm^{-1}		Irrep.
	100 K	150 K	
237	232	232	B_{3g}
258	242	241	A_g
320	309	309	B_{1g}
331	315	315	B_{2g}
381	361	360	A_g
392	369	368	B_{3g}
425	410	410	A_g
437	412	412	B_{1g}
440	418	418	B_{2g}
n. o.	473	474	-
537	520	520	A_g
560	535	535	B_{3g}
584	558	559	A_g
609	578	578	B_{3g}
630	599	601	A_g
653	625	625	B_{1g}
686	644	645	B_{3g}
720	678	677	A_g

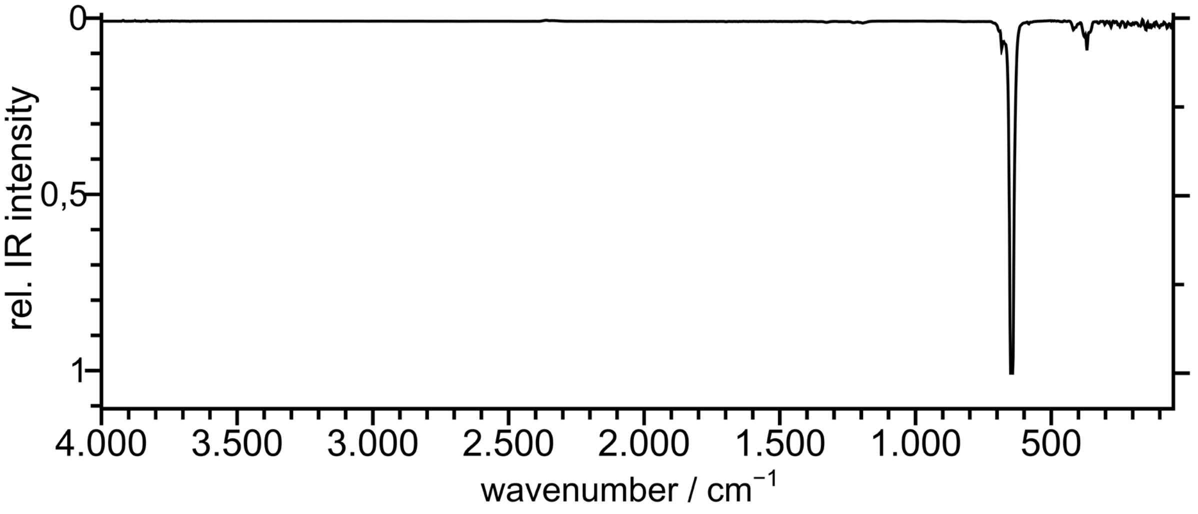

Figure S6: Infrared spectrum of gaseous BrF_5 recorded at room temperature at a total pressure of 10 mbar.

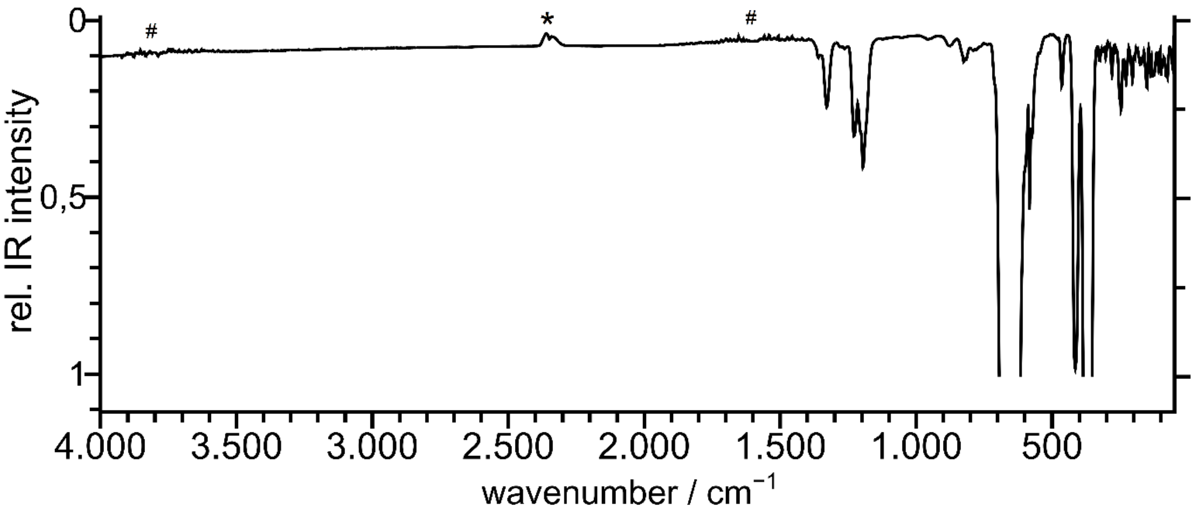

Figure S7: Infrared spectrum of gaseous BrF_5 recorded at room temperature at a total pressure of 125 mbar. The band marked with an asterisk is due to the absorption of atmospheric CO_2, the “noisy” regions marked with a hash are due to the absorption of atmospheric moisture outside the measurement cell.

Table S3: Calculated and observed fundamental vibrations of BrF_5. Raman bands were measured on liquid, IR bands on gaseous BrF_5 at room temperature. Anharmonic frequency calculations were performed at CCSD(T)/cc-pVTZ level of theory using the VPT2 method.

	$\tilde{\nu}$ (calculated) / cm^{-1}	$\tilde{\nu}$ (measured) / cm^{-1}		Irrep.	Description / Comment
		IR	Raman		
ν_1	693	683	687	A_1	stretching vibration $\nu(Br{-}F_{ax})$
ν_2	591	582	569	A_1	symmetric stretching vibration $\nu_s(Br{-}F_{eq})$
ν_3	395	369	365	A_1	out-of-plane bending $\pi(Br{-}F_{eq})$
ν_4	563	-	535	B_1	antisymmetric stretching $\nu_s(Br{-}F_{eq})$
ν_5	243	-	n.o.	B_1	antisymmetric deformation $\delta_a(Br{-}F_{eq})$
ν_6	321	-	312	B_2	symmetric in-plane bending $\delta_s(Br{-}F_{eq})$
ν_7	667	646	n.o.	E	degenerate stretching $\nu_d(Br{-}F_{eq})$
ν_8	427	414	414	E	degenerate out-of-plane bending $\pi_d(Br{-}F_{eq})$
ν_9	245	247	238	E	degenerate in-plane bending $\delta_d(Br{-}F_{eq})$

Table S4: Calculated and observed combination bands for the infrared spectrum of gaseous BrF_5. Anharmonic frequency calculations were performed at the CCSD(T)/cc-pVTZ- level of theory using the VPT2 method. Only modes with non-zero intensity in the quantum chemical calculation are listed

<table>
<tr><th>Anharmonic Frequency / cm^{-1}</th><th>Assignment</th><th>Observed frequency / cm^{-1}</th></tr>
<tr><td>488</td><td>v5+v5</td><td rowspan="2">464</td></tr>
<tr><td>489</td><td>v9+v5</td></tr>
<tr><td>492</td><td>v9+v9</td><td>480</td></tr>
<tr><td>567</td><td>v6+v9</td><td>573</td></tr>
<tr><td>640</td><td>v3+v9</td><td rowspan="2">629</td></tr>
<tr><td>644</td><td>v6+v6</td></tr>
<tr><td>672</td><td>v5+v8</td><td rowspan="2">646</td></tr>
<tr><td>672</td><td>v8+v9</td></tr>
<tr><td>748</td><td>v6+v8</td><td>n.o.</td></tr>
<tr><td>788</td><td>v3+v3</td><td>n.o.</td></tr>
<tr><td>806</td><td>v4+v5</td><td rowspan="2">788</td></tr>
<tr><td>808</td><td>v4+v9</td></tr>
<tr><td>821</td><td>v3+v8</td><td>817</td></tr>
<tr><td>836</td><td>v2+v9</td><td>825</td></tr>
<tr><td>854</td><td>v8+v8</td><td>n.o.</td></tr>
<tr><td>909</td><td>v5+v7</td><td rowspan="2">878</td></tr>
<tr><td>913</td><td>v7+v9</td></tr>
<tr><td>939</td><td>v1+v9</td><td>n.o.</td></tr>
<tr><td>985</td><td>v2+v3</td><td rowspan="3">956</td></tr>
<tr><td>988</td><td>v6+v7</td></tr>
<tr><td>990</td><td>v4+v8</td></tr>
<tr><td>1017</td><td>v2+v8</td><td>n.o.</td></tr>
<tr><td>1061</td><td>v3+v7</td><td>n.o.</td></tr>
<tr><td>1087</td><td>v1+v3</td><td>1056</td></tr>
<tr><td>1094</td><td>v7+v8</td><td>n.o.</td></tr>
<tr><td>1118</td><td>v1+v8</td><td>n.o.</td></tr>
<tr><td>1125</td><td>v4+v4</td><td>n.o.</td></tr>
<tr><td>1181</td><td>v2+v2</td><td>1122</td></tr>
<tr><td>1228</td><td>v4+v7</td><td>1195</td></tr>
<tr><td>1257</td><td>v2+v7</td><td>1228</td></tr>
<tr><td>1280</td><td>v1+v2</td><td>1265</td></tr>
<tr><td>1334</td><td>v7+v7</td><td>n.o.</td></tr>
<tr><td>1360</td><td>v1+v7</td><td>1329</td></tr>
<tr><td>1381</td><td>v1+v1</td><td>1360</td></tr>
</table>

Quantum chemical calculations

Z-Matrices for CFOUR calculations

BrF_5 optimized geometry (CCSD(T)/cc-pVTZ)

```
BR
F 1 R1*
F 1 R2* 2 A1
F 1 R2* 2 A1 3 D90
F 1 R2* 2 A1 3 D180
F 1 R2* 2 A1 3 D270

R1   =      1.704989415306438
R2   =      1.760941493713429
A1   =     84.110820091856013
D90  =      90.000000000000000
D180 =      180.000000000000000
D270 =      -90.000000000000014

*CFOUR(CALC=CCSD(T)
BASIS=SPECIAL
CHARGE=0
SCF_CONV=10
CC_CONV=10
LINEQ_CONV=10
FROZEN_CORE=ON
CC_PROG=ECC
SYMMETRY=ON
ABCDTYPE=AOBASIS
MEMORY_SIZE=10000
MEM_UNIT=MB)

BR:cc-pVTZ
F:cc-pVTZ
F:cc-pVTZ
F:cc-pVTZ
F:cc-pVTZ
F:cc-pVTZ
```

BrF_5 NMRshift calculation (CCSD(T)/cc-pwCVTZ)

```
*CFOUR(CALC=CCSD(T)
BASIS=SPECIAL
CHARGE=0
SCF_DAMPING=500
SCF_MAXCYC=500
SCF_CONV=10
PROP=NMR
TREAT_PERTURBATION=SEQUENTIAL
CC_CONV=10
LINEQ_CONV=10
FROZEN_CORE=ON
CC_PROG=ECC
SYMMETRY=ON
ABCDTYPE=AOBASIS
MEMORY_SIZE=10000
MEM_UNIT=MB)

BR:cc-pwCVTZ
F:cc-pwCVTZ
F:cc-pwCVTZ
F:cc-pwCVTZ
F:cc-pwCVTZ
F:cc-pwCVTZ
```

$CFCl_3$ optimized geometry (CCSD(T)/cc-pVTZ)

```
C
F 1 R1*
CL 1 R2* 2 A110
CL 1 R2* 2 A110 3 D120
CL 1 R2* 2 A110 3 D240

R1   =     1.329791350703937
R2   =     1.775814798198008
A110 =   110.000000000000000
D120 =   120.000000000000014
D240 =  -120.000000000000099

*CFOUR(CALC=CCSD(T)
BASIS=SPECIAL
CHARGE=0
SCF_CONV=10
CC_CONV=10
LINEQ_CONV=10
FROZEN_CORE=ON
CC_PROG=ECC
SYMMETRY=ON
ABCDTYPE=AOBASIS
MEMORY_SIZE=10000
MEM_UNIT=MB)

C:cc-pVTZ
F:cc-pVTZ
CL:cc-pVTZ
CL:cc-pVTZ
CL:cc-pVTZ
```

$CFCl_3$ NMRshift calculation (CCSD(T)/cc-pwCVTZ)

```
*CFOUR(CALC=CCSD(T)
BASIS=SPECIAL
CHARGE=0
SCF_CONV=10
SCF_DAMPING=500
SCF_MAXCYC=200
PROP=NMR
TREAT_PERTURBATION=SEQUENTIAL
CC_CONV=10
LINEQ_CONV=10
FROZEN_CORE=ON
CC_PROG=ECC
SYMMETRY=ON
ABCDTYPE=AOBASIS
MEMORY_SIZE=10000
MEM_UNIT=MB)

C:cc-pwCVTZ
F:cc-pwCVTZ
CL:cc-pwCVTZ
CL:cc-pwCVTZ
CL:cc-pwCVTZ
```

COF_3^- optimized geometry (CCSD(T)/cc-pVTZ)

```
C
O 1 R1*
F 1 R2* 2 A110
F 1 R2* 2 A110 3 D120
F 1 R2* 2 A110 3 D240

R1   =     1.248988205039689
R2   =     1.401344745548687
A110 =   110.000000000000000
D120 =   120.000000000000099
D240 =  -120.000000000000142

*CFOUR(CALC=CCSD(T)
BASIS=SPECIAL
CHARGE=-1
GEO_CONV=8
SCF_DAMPING=500
SCF_CONV=10
CC_CONV=10
LINEQ_CONV=10
FROZEN_CORE=ON
CC_PROG=ECC
SYMMETRY=ON
ABCDTYPE=AOBASIS
MEMORY_SIZE=8000
MEM_UNIT=MB)

C:cc-pVTZ
O:cc-pVTZ
F:cc-pVTZ
F:cc-pVTZ
F:cc-pVTZ
```

COF_2 optimized geometry (CCSD(T)/cc-pVTZ)

```
C
O 1 R1*
F 1 R2* 2 A1*
F 1 R2* 2 A1* 3 A2*

R1   =     1.176040896059793
R2   =     1.313347915797420
A1   =   126.082497510162995
A2   =   180.000000000000000

*CFOUR(CALC=CCSD(T)
BASIS=SPECIAL
CHARGE=0
GEO_CONV=8
SCF_DAMPING=500
SCF_CONV=10
CC_CONV=10
LINEQ_CONV=10
FROZEN_CORE=ON
CC_PROG=ECC
SYMMETRY=ON
ABCDTYPE=AOBASIS
MEMORY_SIZE=8000
MEM_UNIT=MB)

C:cc-pVTZ
O:cc-pVTZ
F:cc-pVTZ
F:cc-pVTZ
```

$[BrF_6]^-$ optimized geometry (CCSD(T)/cc-pVTZ)

```
BR
F 1 R*
F 1 R* 2 A
F 1 R* 3 A 2 T1
F 1 R* 4 A 3 T2
F 1 R* 5 A 4 T1
F 1 R* 6 A 5 T2

R    =     1.872555153029294
A    =    90.000000000000000
T1   =    90.000000000000000
T2   =   -90.000000000000014

*CFOUR(CALC=CCSD(T)
BASIS=SPECIAL
CHARGE=-1
GEO_CONV=8
SCF_DAMPING=500
SCF_CONV=10
CC_CONV=10
LINEQ_CONV=10
FROZEN_CORE=ON
CC_PROG=ECC
SYMMETRY=ON
ABCDTYPE=AOBASIS
MEMORY_SIZE=8000
MEM_UNIT=MB)

BR:cc-pVTZ
F:cc-pVTZ
F:cc-pVTZ
F:cc-pVTZ
F:cc-pVTZ
F:cc-pVTZ
F:cc-pVTZ
```

Employed *k*-point meshes

Table S5. The Monkhorst-Pack-type k-point meshes used in the solid-state calculations with CRYSTAL17.

Structure	*k*-mesh
LT-BrF_5	6×6×6
HT-BrF_5	4×6×6
$RbBrF_6$	6×6×6
$KBrF_6$	6×6×6
BrF_5, *Cmc*2_1	6×6×6
BrF_5, hypothetical AuF_5-type	4×2×8

Optimized geometries of the BrF_5 crystal structures in CIF format

The CIFs below have been standardized by FINDSYM (https://stokes.byu.edu/iso/findsym.php) after the geometry optimization.

LT-BrF_5 with DFT-PBE0-D3

```
data_findsym-output
_audit_creation_method FINDSYM

_cell_length_a    6.3851044900
_cell_length_b    7.2718902300
_cell_length_c    7.8449227400
_cell_angle_alpha 90.0000000000
_cell_angle_beta  96.9947170000
_cell_angle_gamma 90.0000000000
_cell_volume      361.5427182281

_symmetry_space_group_name_H-M "P 1 21/c 1"
_symmetry_Int_Tables_number 14
_space_group.reference_setting '014:-P 2ybc'
_space_group.transform_Pp_abc a,b,c;0,0,0

loop_
_space_group_symop_id
_space_group_symop_operation_xyz
1 x,y,z
2 -x,y+1/2,-z+1/2
3 -x,-y,-z
4 x,-y+1/2,z+1/2

loop_
_atom_site_label
_atom_site_type_symbol
_atom_site_symmetry_multiplicity
_atom_site_Wyckoff_label
_atom_site_fract_x
_atom_site_fract_y
_atom_site_fract_z
_atom_site_occupancy
_atom_site_fract_symmform
Br1 Br   4 e 0.28720 0.07185 0.25595 1.00000 Dx,Dy,Dz
F1  F    4 e 0.20703 0.89877 0.11698 1.00000 Dx,Dy,Dz
F2  F    4 e 0.24644 0.21306 0.06840 1.00000 Dx,Dy,Dz
F3  F    4 e 0.31129 0.89693 0.41337 1.00000 Dx,Dy,Dz
F4  F    4 e 0.01710 0.09030 0.27780 1.00000 Dx,Dy,Dz
F5  F    4 e 0.54233 0.01289 0.20385 1.00000 Dx,Dy,Dz

# end of cif
```

LT-BrF_5 with DFT-PBE0

```
data_findsym-output
_audit_creation_method FINDSYM


_cell_length_a    8.3782527400
_cell_length_b    6.2899267800
_cell_length_c    8.7197017800
_cell_angle_alpha 90.0000000000
_cell_angle_beta  100.2877110000
_cell_angle_gamma 90.0000000000
_cell_volume      452.1285722618

_symmetry_space_group_name_H-M "P 1 21/c 1"
_symmetry_Int_Tables_number 14
_space_group.reference_setting '014:-P 2ybc'
_space_group.transform_Pp_abc a,b,c;0,0,0

loop_
_space_group_symop_id
_space_group_symop_operation_xyz
1 x,y,z
2 -x,y+1/2,-z+1/2
3 -x,-y,-z
4 x,-y+1/2,z+1/2

loop_
_atom_site_label
_atom_site_type_symbol
_atom_site_symmetry_multiplicity
_atom_site_Wyckoff_label
_atom_site_fract_x
_atom_site_fract_y
_atom_site_fract_z
_atom_site_occupancy
_atom_site_fract_symmform
Br1 Br   4 e 0.29318 0.09267  0.19856 1.00000 Dx,Dy,Dz
F1  F    4 e 0.20156 -0.06547 0.04995 1.00000 Dx,Dy,Dz
F2  F    4 e 0.31663 0.27029  0.04414 1.00000 Dx,Dy,Dz
F3  F    4 e 0.25066 0.88250  0.31820 1.00000 Dx,Dy,Dz
F4  F    4 e 0.09732 0.19454  0.19561 1.00000 Dx,Dy,Dz
F5  F    4 e 0.47320 -0.04454 0.17141 1.00000 Dx,Dy,Dz

# end of cif
```

HT-BrF_5 with DFT-PBE0-D3

```
data_findsym-output
_audit_creation_method FINDSYM

_cell_length_a    8.0907523800
_cell_length_b    6.4653327700
_cell_length_c    7.0584331600
_cell_angle_alpha 90.0000000000
_cell_angle_beta  90.0000000000
_cell_angle_gamma 90.0000000000
_cell_volume      369.2224493939

_symmetry_space_group_name_H-M "P 21/n 21/m 21/a"
_symmetry_Int_Tables_number 62
_space_group.reference_setting '062:-P 2ac 2n'
_space_group.transform_Pp_abc a,b,c;0,0,0

loop_
_space_group_symop_id
_space_group_symop_operation_xyz
1 x,y,z
2 x+1/2,-y+1/2,-z+1/2
3 -x,y+1/2,-z
4 -x+1/2,-y,z+1/2
5 -x,-y,-z
6 -x+1/2,y+1/2,z+1/2
```

7 x,-y+1/2,z
8 x+1/2,y,-z+1/2

loop_
_atom_site_label
_atom_site_type_symbol
_atom_site_symmetry_multiplicity
_atom_site_Wyckoff_label
_atom_site_fract_x
_atom_site_fract_y
_atom_site_fract_z
_atom_site_occupancy
_atom_site_fract_symmform
Br1 Br 4 c 0.74461 0.25000 0.08115 1.00000 Dx,0,Dz
F1 F 4 c 0.61593 0.25000 0.89029 1.00000 Dx,0,Dz
F2 F 4 c -0.09564 0.25000 -0.08721 1.00000 Dx,0,Dz
F3 F 4 c 0.55500 0.25000 0.21356 1.00000 Dx,0,Dz
F4 F 8 d 0.73176 -0.02228 0.05929 1.00000 Dx,Dy,Dz

end of cif

HT-BrF_5 with DFT-PBE0

data_findsym-output
_audit_creation_method FINDSYM

_cell_length_a 8.1536304100
_cell_length_b 7.1010546700
_cell_length_c 7.3524818300
_cell_angle_alpha 90.0000000000
_cell_angle_beta 90.0000000000
_cell_angle_gamma 90.0000000000
_cell_volume 425.7041048644

_symmetry_space_group_name_H-M "P 21/n 21/m 21/a"
_symmetry_Int_Tables_number 62
_space_group.reference_setting '062:-P 2ac 2n'
_space_group.transform_Pp_abc a,b,c;0,0,0

loop_
_space_group_symop_id
_space_group_symop_operation_xyz
1 x,y,z
2 x+1/2,-y+1/2,-z+1/2
3 -x,y+1/2,-z
4 -x+1/2,-y,z+1/2
5 -x,-y,-z
6 -x+1/2,y+1/2,z+1/2
7 x,-y+1/2,z
8 x+1/2,y,-z+1/2

loop_
_atom_site_label
_atom_site_type_symbol
_atom_site_symmetry_multiplicity
_atom_site_Wyckoff_label
_atom_site_fract_x
_atom_site_fract_y
_atom_site_fract_z
_atom_site_occupancy
_atom_site_fract_symmform
Br1 Br 4 c 0.74634 0.25000 0.08467 1.00000 Dx,0,Dz
F1 F 4 c 0.61420 0.25000 -0.09463 1.00000 Dx,0,Dz
F2 F 4 c -0.09967 0.25000 -0.08231 1.00000 Dx,0,Dz
F3 F 4 c 0.56138 0.25000 0.21759 1.00000 Dx,0,Dz
F4 F 8 d 0.73295 0.00199 0.06528 1.00000 Dx,Dy,Dz

end of cif

BrF_5 *Cmc*2_1 with DFT-PBE0-D3

```
data_findsym-output
_audit_creation_method FINDSYM

_cell_length_a    6.4947502347
_cell_length_b    7.1668083054
_cell_length_c    8.0184053900
_cell_angle_alpha 90.0000000000
_cell_angle_beta  90.0000000000
_cell_angle_gamma 90.0000000000
_cell_volume      373.2297482664

_symmetry_space_group_name_H-M "C m c 21"
_symmetry_Int_Tables_number 36
_space_group.reference_setting '036:C 2c -2'
_space_group.transform_Pp_abc a,b,c;0,0,0

loop_
_space_group_symop_id
_space_group_symop_operation_xyz
1 x,y,z
2 -x,-y,z+1/2
3 -x,y,z
4 x,-y,z+1/2
5 x+1/2,y+1/2,z
6 -x+1/2,-y+1/2,z+1/2
7 -x+1/2,y+1/2,z
8 x+1/2,-y+1/2,z+1/2

loop_
_atom_site_label
_atom_site_type_symbol
_atom_site_symmetry_multiplicity
_atom_site_Wyckoff_label
_atom_site_fract_x
_atom_site_fract_y
_atom_site_fract_z
_atom_site_occupancy
_atom_site_fract_symmform
Br1 Br   4 a 0.00000 0.82959  0.24936 1.00000 0,Dy,Dz
F1  F    4 a 0.00000 0.64749  0.38572 1.00000 0,Dy,Dz
F2  F    4 a 0.00000 0.65656  0.09450 1.00000 0,Dy,Dz
F3  F    4 a 0.00000 -0.03284 0.43688 1.00000 0,Dy,Dz
F4  F    8 b 0.22893 0.30888  0.26177 1.00000 Dx,Dy,Dz

# end of cif
```

Hypothetical BrF_5 structure in AuF_5 structure type with DFT-PBE0-D3

```
data_findsym-output
_audit_creation_method FINDSYM

_cell_length_a    9.4659911600
_cell_length_b    15.3694904600
_cell_length_c    4.9455023900
_cell_angle_alpha 90.0000000000
_cell_angle_beta  90.0000000000
_cell_angle_gamma 90.0000000000
_cell_volume      719.5085852402

_symmetry_space_group_name_H-M "P 21/n 21/m 21/a"
_symmetry_Int_Tables_number 62
_space_group.reference_setting '062:-P 2ac 2n'
_space_group.transform_Pp_abc a,b,c;0,0,0

loop_
_space_group_symop_id
_space_group_symop_operation_xyz
1 x,y,z
2 x+1/2,-y+1/2,-z+1/2
3 -x,y+1/2,-z
4 -x+1/2,-y,z+1/2
5 -x,-y,-z
6 -x+1/2,y+1/2,z+1/2
7 x,-y+1/2,z
8 x+1/2,y,-z+1/2

loop_
_atom_site_label
_atom_site_type_symbol
_atom_site_symmetry_multiplicity
_atom_site_Wyckoff_label
_atom_site_fract_x
_atom_site_fract_y
_atom_site_fract_z
_atom_site_occupancy
_atom_site_fract_symmform
Br1 Br   8 d 0.13392  0.85668  0.56297 1.00000 Dx,Dy,Dz
F1  F    8 d 0.25260  0.86319  0.84121 1.00000 Dx,Dy,Dz
F2  F    4 c 0.75297  0.25000  0.63221 1.00000 Dx,0,Dz
F3  F    4 c -0.01443 0.25000  0.24870 1.00000 Dx,0,Dz
F4  F    8 d 0.01727  0.87053  0.28403 1.00000 Dx,Dy,Dz
F5  F    8 d 0.23983  -0.06764 0.40939 1.00000 Dx,Dy,Dz
F6  F    8 d 0.03730  -0.06430 0.72320 1.00000 Dx,Dy,Dz

# end of cif
```

Optimized geometries of the K[BrF$_6$] and Rb[BrF$_6$] crystal structures in CIF format

The CIFs below have been standardized by FINDSYM (https://stokes.byu.edu/iso/findsym.php) after the geometry optimization.

K[BrF$_6$]

```
data_findsym-output
_audit_creation_method FINDSYM

_cell_length_a    7.4871537070
_cell_length_b    7.4871537070
_cell_length_c    7.4052270844
_cell_angle_alpha 90.0000000000
_cell_angle_beta  90.0000000000
_cell_angle_gamma 120.0000000000
_cell_volume      359.5029932094

_symmetry_space_group_name_H-M "R -3 (hexagonal axes)"
_symmetry_Int_Tables_number 148
_space_group.reference_setting '148:-R 3'
_space_group.transform_Pp_abc a,b,c;0,0,0

loop_
_space_group_symop_id
_space_group_symop_operation_xyz
1 x,y,z
2 -y,x-y,z
3 -x+y,-x,z
4 -x,-y,-z
5 y,-x+y,-z
6 x-y,x,-z
7 x+1/3,y+2/3,z+2/3
8 -y+1/3,x-y+2/3,z+2/3
9 -x+y+1/3,-x+2/3,z+2/3
10 -x+1/3,-y+2/3,-z+2/3
11 y+1/3,-x+y+2/3,-z+2/3
12 x-y+1/3,x+2/3,-z+2/3
13 x+2/3,y+1/3,z+1/3
14 -y+2/3,x-y+1/3,z+1/3
15 -x+y+2/3,-x+1/3,z+1/3
16 -x+2/3,-y+1/3,-z+1/3
17 y+2/3,-x+y+1/3,-z+1/3
18 x-y+2/3,x+1/3,-z+1/3

loop_
_atom_site_label
_atom_site_type_symbol
_atom_site_symmetry_multiplicity
_atom_site_Wyckoff_label
_atom_site_fract_x
_atom_site_fract_y
_atom_site_fract_z
_atom_site_occupancy
_atom_site_fract_symmform
Br1 Br   3 a 0.00000 0.00000  0.00000 1.00000 0,0,0
K1  K    3 b 0.00000 0.00000  0.50000 1.00000 0,0,0
F1  F   18 f 0.77660 -0.06068 0.14972 1.00000 Dx,Dy,Dz

# end of cif
```

Rb[BrF$_6$]

```
data_findsym-output
_audit_creation_method FINDSYM

_cell_length_a    7.6730367765
_cell_length_b    7.6730367765
_cell_length_c    7.7378972244
_cell_angle_alpha 90.0000000000
_cell_angle_beta  90.0000000000
_cell_angle_gamma 120.0000000000
_cell_volume      394.5373727795

_symmetry_space_group_name_H-M "R -3 (hexagonal axes)"
_symmetry_Int_Tables_number 148
_space_group.reference_setting '148:-R 3'
_space_group.transform_Pp_abc a,b,c;0,0,0

loop_
_space_group_symop_id
_space_group_symop_operation_xyz
1 x,y,z
2 -y,x-y,z
3 -x+y,-x,z
4 -x,-y,-z
5 y,-x+y,-z
6 x-y,x,-z
7 x+1/3,y+2/3,z+2/3
8 -y+1/3,x-y+2/3,z+2/3
9 -x+y+1/3,-x+2/3,z+2/3
10 -x+1/3,-y+2/3,-z+2/3
11 y+1/3,-x+y+2/3,-z+2/3
12 x-y+1/3,x+2/3,-z+2/3
13 x+2/3,y+1/3,z+1/3
14 -y+2/3,x-y+1/3,z+1/3
15 -x+y+2/3,-x+1/3,z+1/3
16 -x+2/3,-y+1/3,-z+1/3
17 y+2/3,-x+y+1/3,-z+1/3
18 x-y+2/3,x+1/3,-z+1/3

loop_
_atom_site_label
_atom_site_type_symbol
_atom_site_symmetry_multiplicity
_atom_site_Wyckoff_label
_atom_site_fract_x
_atom_site_fract_y
_atom_site_fract_z
_atom_site_occupancy
_atom_site_fract_symmform
Rb1 Rb   3 b 0.00000 0.00000 0.50000 1.00000 0,0,0
Br1 Br   3 a 0.00000 0.00000 0.00000 1.00000 0,0,0
F1  F   18 f 0.78204 0.83794 0.14222 1.00000 Dx,Dy,Dz

# end of cif
```

Calculated Raman spectra of LT-BrF_5 and HT-BrF_5

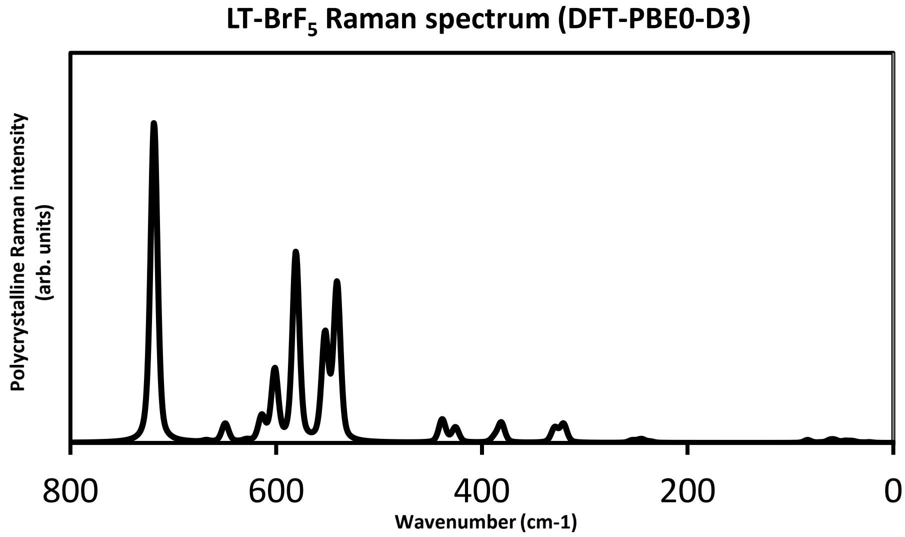

Figure S8. The Raman spectrum of LT-BrF_5 calculated with DFT-PBE0-D3.

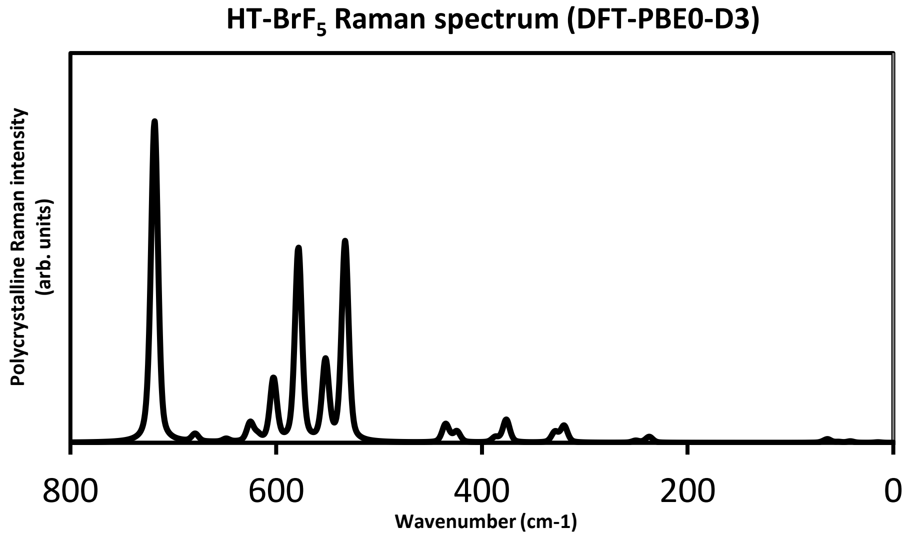

Figure S9. The Raman spectrum of HT-BrF_5 calculated with DFT-PBE0-D3.

Rietveld refinement details

Table S6. Selected crystallographic data and details of the Rietveld refinement on powder X-ray diffraction data of BrF_5.

Formula	BrF_5
Molar mass / g·mol^{-1}	174.91
Space group (No.)	*Pnma* (62)
a / Å	7.8412(5)
b / Å	6.4252(6)
c / Å	7.2683(6)
V / Å^3	366.19(5)
Z	4
Pearson symbol	*oP*24
$\rho_{calc.}$ / g·cm^{-3}	3.172
Color of the powder	colorless
T / K	180
λ / Å	1.54060 (Cu-$K_{\alpha1}$)
$2\theta_{min}$, $2\theta_{max}$, $2\theta_{step}$ / °	15.000, 71.265, 0.015
No. of data points	3752
Refined parameters	90[a]
Background parameters	12
No. of restraints	0
No. of constraints	0
Peak shape function	TCHZ
Background	Chebyshev polynomial
S	1.295
R_p, R_{wp}	3.773, 5.416
R_p*, R_{wp}*	19.154, 14.723
$R_B(I)$	1.788

* Background-corrected *R*-factors. [a] Refined parameters include spherical harmonics of order 8 and the *Le Bail* and *Jouanneaux* approach for anisotropic peak half-width and shape.

Crystallographic considerations

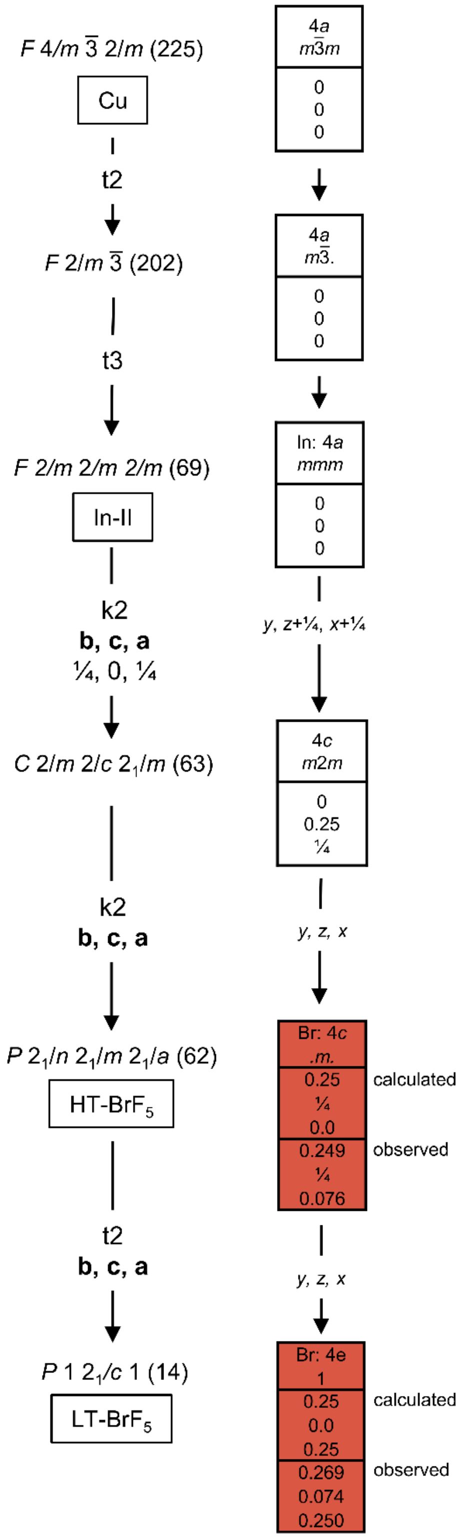

Figure S10. Bärnighausen tree showing the group-subgroup relations of the Cu type with the HT- and LT-modification of BrF_5.

Variable-temperature powder X-ray diffraction on BrF_5

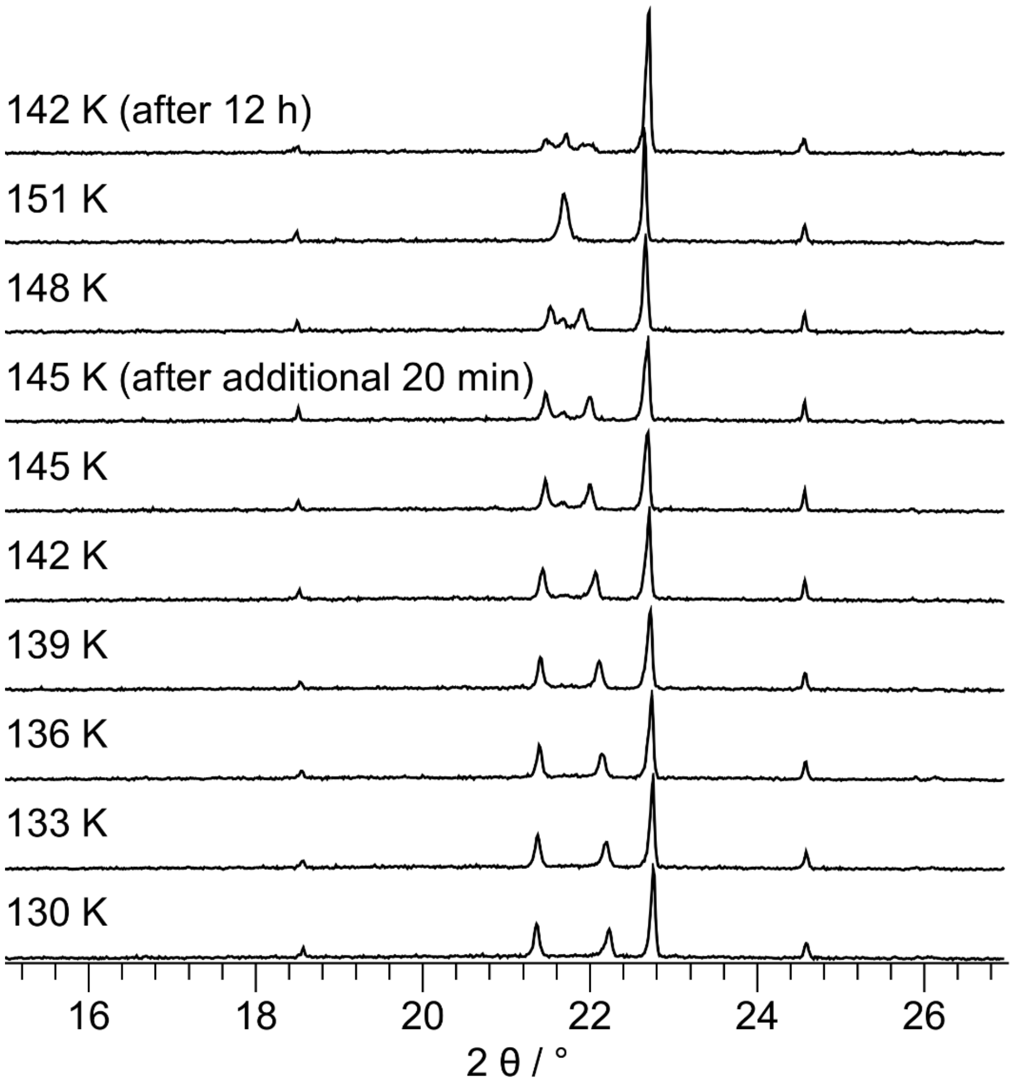

Figure S 11. Powder X-ray diffraction patterns of BrF_5 recorded at different temperatures using Cu-$K_{\alpha1}$ radiation. To narrow down the temperature of the phase-change from LT- to HT-BrF_5 only the 2θ-range from 15 to 27° 2θ was measured as the differences of the powder patterns are most pronounced in that region. Stepwise warming was used to avoid hysteresis effects which usually occur upon cooling. BrF_5 was cooled to 130 K and only the crystalline phase of LT-BrF_5 was present. Each powder pattern was recorded within 3 minutes. After each temperature change, we waited 10 minutes and recorded the patterns. Longer waiting times are given in the Figure.

Author Contributions

Martin Möbs: Planning and conducting the experiments, main data acquisition and interpretation, manuscript preparation.

Tim Graubner: Quantum chemical calculations and evaluation of vibrational, NMR spectra and $MBrF_6$ solid-state calculation, manuscript preparation.

Kim Eklund: Quantum-chemical solid-state calculations, structure prediction and evaluation, manuscript preparation.

Antti J. Karttunen: Quantum chemical calculation, CRYSTAL17 basis set development, manuscript preparation.

Florian Kraus: Project supervision, manuscript preparation.
